# Heterologous production of tungsten-dependent formate dehydrogenase I from *Methylorubrum extorquens* in *Escherichia coli* reveals α-subunit maturation as the major bottleneck

**DOI:** 10.1186/s40643-026-01092-7

**Published:** 2026-07-24

**Authors:** Ngoc Minh Chau Nguyen, Huichang Ryu, Joon Young Park, Yong Hwan Kim, Sunghoon Park

**Affiliations:** 1 School of Energy and Chemical Engineering, UNIST, 50, UNIST-gil, Eonyang-eup, Ulju-gun, Ulsan, 44919 Republic of Korea; 2 Korea Research Institute of Chemical Technology, KRICT, Daejeon, Republic of Korea

**Keywords:** *Methylorubrum extorquens*, Tungsten-dependent formate dehydrogenase, W-bis-MGD, Tungstate transport, α-subunit maturation, Post-translational instability

## Abstract

*Methylorubrum extorquens* formate dehydrogenase I (Me-FDH1) is a tungsten-dependent heterodimeric enzyme with high activity for CO_2_/formate interconversion, making it an attractive biocatalyst for carbon capture, formate production, and bioelectrocatalysis. Its broader application, however, is limited by the lack of a heterologous production host for this complex metalloenzyme. Here, we systematically evaluated *Escherichia coli* as a host for Me-FDH1 production and compared its performance with that of the native host and a previous heterologous host. Across multiple *E. coli* strains, active Me-FDH1 was obtained only when tungstate uptake was supported by a functional *Mod*ABC system or by heterologous expression of the *Tup*BCA transporter, demonstrating that *E. coli* can synthesize and incorporate the W-bis-MGD cofactor. Nevertheless, expression remained low (< 1% of total cellular protein), and the purified enzyme displayed only 4–14 U mg^− 1^ specific activity, far below the 80–100 U.mg^− 1^ observed for the enzyme produced in *M. extorquens*. Operon redesign, altered gene order, stronger ribosome-binding sites, SUMO fusion, chaperone co-expression, and codon harmonization did not improve α-subunit production. In both *E. coli* and *M. extorquens*, the α-subunit was poorly produced in the absence of the β-subunit, indicating that the β-subunit contributes to α-subunit stabilization and/or maturation. Cell-free translation produced both subunits efficiently, showing that the principal barrier in *E. coli* is not transcription or translation, but post-translational instability and likely proteolytic loss of the α-subunit. These findings define the key bottleneck for Me-FDH1 production in *E. coli* and provide a roadmap for engineering hosts for tungsten-containing enzymes.

**Graphical abstract:**

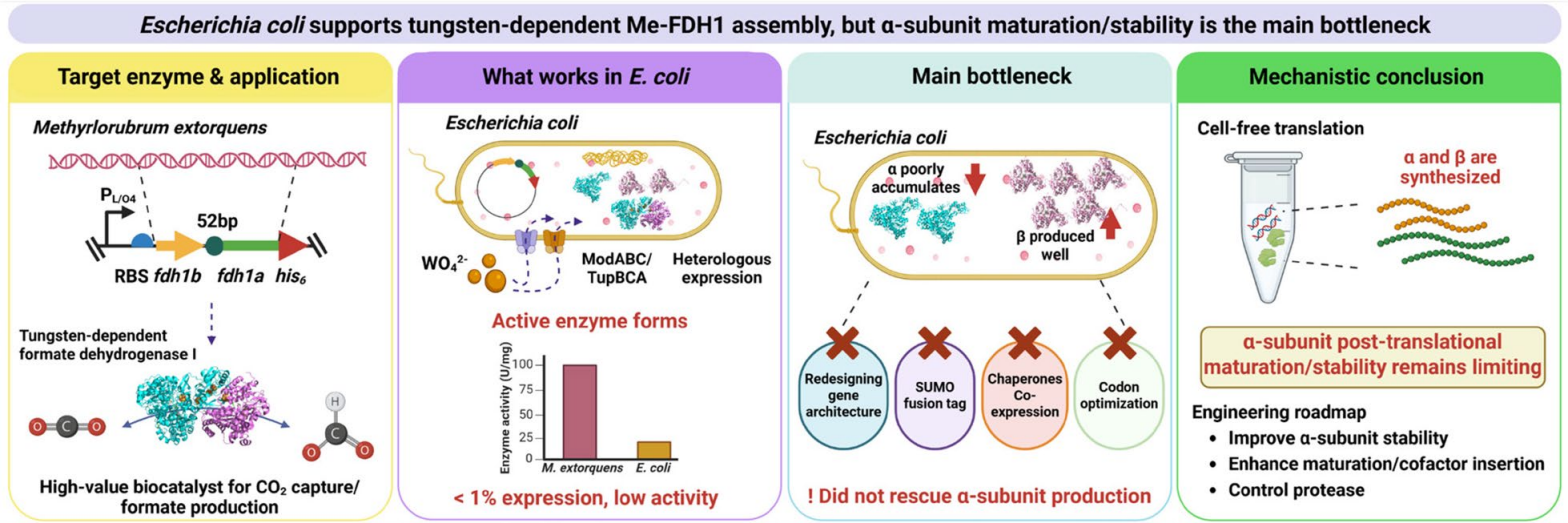

**Supplementary Information:**

The online version contains supplementary material available at 10.1186/s40643-026-01092-7.

## Key points

E. coli produced active Me-FDH1 when tungstate uptake was enabled.

ModABC or TupBCA supported W-bis-MGD-dependent Me-FDH1 activity.

The α-subunit was the major bottleneck in Me-FDH1 production.

The β-subunit promoted α-subunit accumulation and/or maturation.

Cell-free translation revealed post-translational loss of the α-subunit.

**Supplementary Information:**

The online version contains supplementary material available at 10.1186/s40643-026-01092-7.

## Introduction

The enzymatic reduction of carbon dioxide to formate is an attractive route for carbon capture and utilization because formate can serve both as a C1 chemical feedstock and as a liquid hydrogen carrier (Jang et al. 2018). Formate dehydrogenases (FDHs) are therefore important biocatalysts for sustainable CO_2_ conversion. Among them, formate dehydrogenase I from *Methylorubrum extorquens* (Me-FDH1) is particularly attractive because it efficiently catalyzes the interconversion of CO_2_ and formate and also supports direct electron-transfer bioelectrocatalysis, making it relevant to enzymatic and electrochemical carbon-recycling systems (Laukel et al. 2003; Jang et al. 2018; Yoshikawa et al. 2022).

Me-FDH1 is unusual among FDHs in that it contains a tungsten cofactor rather than the more common molybdenum cofactor (Park et al. 2024). The enzyme is a heterodimer composed of an approximately 107 kDa α-subunit and a 62 kDa β-subunit. The α-subunit carries a tungsto-bis(molybdopterin guanine dinucleotide) cofactor (W-bis-MGD), whereas multiple Fe-S clusters are distributed across both subunits (Yoshikawa et al. 2022). This complex architecture likely underlies the high catalytic performance of Me-FDH1, but it also makes heterologous production demanding because active holoenzyme formation requires coordinated expression of two large subunits, correct folding, W-bis-MGD biosynthesis and insertion, and proper Fe-S cluster assembly.

Me-FDH1 can be produced efficiently in its native host, *M. extorquens*, where recombinant expression under a strong promoter yields high activity and high intracellular accumulation (Jang et al. 2018; Ryu et al. 2024). However, homologous production in *M. extorquens* is not ideal for large-scale application because the organism grows relatively slowly, is less convenient for high-cell-density cultivation, and relies on less convenient carbon sources such as methanol or succinate (Ryu et al. 2024, 2025). More recently, *Cupriavidus necator* H16 was shown to be an effective heterologous host, yielding recombinant Me-FDH1 with near-native activity and high volumetric productivity (Ryu et al. 2024, 2025). Nevertheless, recombinant Me-FDH1 still underwent degradation during later cultivation, and deeper host engineering is less straightforward than in *Escherichia coli* (Park et al. 2024; Ryu et al. 2024, 2025).

These considerations make *E. coli* an attractive candidate host. *E. coli* grows rapidly, reaches high cell density, and offers unmatched genetic accessibility. The key question, however, is whether *E. coli* can be adapted to produce a catalytically competent tungsten-containing metalloenzyme as complex as Me-FDH1. In this study, we systematically evaluated *E. coli* as a host for Me-FDH1 production and used this analysis to identify the dominant bottleneck. We tested multiple *E. coli* strains with different tungstate/molybdate transport capacities, examined operon architecture using a series of recombinant plasmids, and evaluated SUMO fusion, chaperone co-expression, and codon harmonization as strategies to improve α-subunit production. We further analyzed transcript levels by RT-PCR and used a cell-free translation system to distinguish between failure of α-subunit synthesis and loss of the α-subunit after synthesis. By comparing the results obtained in *E. coli* with those from *M. extorquens* and a previously developed heterologous host, we aimed to define the host functions that must be engineered to support robust production of Me-FDH1 and related tungsten-dependent enzymes.

## Materials and methods

### Materials

Various *E. coli* strains, including B, BL21 (DE3), BL21 Star™ (DE3) pLysS, BW25113, C ATCC8739, JM109 (DE3), K-12 MG1655, K-12 Shuffle^®^ T7, MC1061, W, W3110, XL-1 Blue and Rosetta 2 (DE3) and a derivative of *M. extorquens* PA1 lacking all four native FDH genes (MeP4; Δ*fdh*1–4) were used in this study (Table 1). *E. coli* DH5α (Toyobo, Japan) was used for gene cloning. DNA sequencing and primer synthesis were performed by Macrogen (Korea). In-Fusion^®^ Snap Assembly Master Mix (TaKaRa, Japan), restriction endonucleases and T4 DNA ligase (New England Biolabs, USA), and Pfu-X DNA polymerase with standard PCR reagents (SolGent, Korea) were used for plasmid construction. Plasmids and genomic DNA were prepared with commercial kits according to the manufacturers’ instructions. Isopropyl-β-D-thiogalactopyranoside (IPTG; Bio Basic, Canada) was used for protein induction, and all other reagents were of analytical grade unless otherwise noted.

**Table 1:** Plasmids and strains used in this study

Plasmids	Description	Source
pCM2	pCM110-P_L/O4_-RBS_b_-*fdh1*ba-His_6_-T7_ter_, Tet^R^	(Ryu et al. 2024)
pCM3	pCM110-P_L/O4_- RBS_b_- *fdh1*a-His_6_- *fdh1*b-T7_ter_, Tet^R^	This study
pCM4	pCM110-P_L/O4_- RBS_b_- *fdh1*b- RBS_b_-*fdh1*a-His_6-_T7_ter_, Tet^R^	This study
pCM5	pCM110-P_L/O4_- RBS_b_-His_6_- *fdh1*b-T7_ter_-P_L/O4_- RBS_b_- *fdh1*a-His_6_-T7_ter_, Tet^R^	This study
pCM6	pCM110-P_L/O4_- RBS_b_-His6- *fdh1*b-T7_ter_, Tet^R^	This study
pCM7	pCM110-P_L/O4_- RBS_b_- *fdh1*a-His6-T7_ter_, Tet^R^	This study
pAC1	pACYC184, p15A ColE1 ori, Cm^R^, Tet^R^	Addgene
pAC2	pAC-P_tac_-RBS-*tup*BCA-T7_ter_, Cm^R^	This study
pRSF1	pRSF-Duet-1, RSF1030 ori, P_T7_, Kan^R^	Addgene
pRSF2	pRSF-P_T7_-*fdh1*ba-His_6_-T7_ter_, Kan^R^	This study
pRSF3	pRSF-P_T7_- *fdh1*a-His_6_-T7_ter_, Kan^R^	This study
pRSF4	pRSF-P_T7_- *fdh1*b-His_6_-T7_ter_, Kan^R^	This study
pRSF5	pRSF-P_T7_- *fdh1*b-His_6_-T7_ter_-P_T7_- *fdh1*a-His_6_-T7_ter_, Kan^R^	This study
pRSF6	pRSF-P_T7_-SUMO- *fdh1*ba-His_6_-T7_ter_, Kan^R^	This study
pRSF7	pRSF-P_T7_-SUMO- *fdh1*a-His_6_-T7_ter_, Kan^R^	This study
pRSF8	pRSF-P_T7_- *fdh1*a codon optimized-His_6_-T7_ter_, Kan^R^	This study
pET1	pET-Duet-1, pBR322-derived ColE1 ori, P_T7_, Am^R^	Addgene
pET2	pET-P_T7_- *fdh1*b codon optimized-His_6_-T7_ter_, Am^R^	This study
pG-KJE8	P_araBAD_-*dnaK*-*dnaJ*-*grpE*, P_lac_-*groES*-*groEL*, Cm^R^, p15A ori	TaKaRa, Japan
pG-Tf2	P_lac_-*tig*, P_lac_-*groES*-*groEL*, Cm^R^, p15A ori	TaKaRa, Japan

Strains	Description	Source
EcB	*E. coli* B wildtype; B-lineage strain included in the host-background screen	DSMZ
EcBL	*E. coli* BL21 (DE3) wildtype; B-derived T7 expression host carrying λDE3, deficient in Lon and OmpT proteases; also used for TupBCA transporter complementation	(Nguyen-Vo et al. 2020)
EcBLP	*E. coli* BL21 Star™ (DE3) pLysS wildtype; T7 expression host carrying *rne*131 for enhanced mRNA stability and pLysS for basal-expression repression	New England Biolabs
EcBW	*E. coli* BW25113 wildtype; K-12 derivative and parent strain of the Keio knockout collection	(Sundara Sekar et al. 2016)
EcC	*E. coli* C ATCC8739 wildtype; C-lineage strain included in the host-background screen	(Sundara Sekar et al. 2016)
*E. coli* DH5α	Cloning host	Toyobo, Japan
EcJM	*E. coli* JM109 (DE3) wildtype; K-12 derivative carrying λDE3 for T7-driven expression	Promega, Korea
EcK	*E. coli* K-12 MG1655 wildtype; standard K-12 reference strain used for initial expression, operon-architecture analysis, and chaperone co-expression tests	(Nguyen-Vo et al. 2020)
EcKT	*E. coli* K-12 Shuffle^®^ T7 wildtype; redox-engineered T7-compatible strain with Δ*trx*B/Δ*gor* and cytoplasmic DsbC, used for SUMO-fusion and codon-harmonization tests	New England Biolabs
EcMC	*E. coli* MC1061 wildtype; K-12 derivative previously used for heterologous expression of the Mo-dependent *Rhodobacter capsulatus* FDH	Thermo Fisher Scientific; (Böhmer et al. 2014; Hartmann and Leimkühler 2013)
EcWA	*E. coli* W ATCC 9637 wildtype; W-lineage strain included in the host-background screen	(Nguyen-Vo et al. 2020)
EcW	*E. coli* W3110 wildtype; K-12-lineage strain included in the host-background screen	ATCC
EcXL	*E. coli* XL-1 Blue wildtype; K-12 derivative with cloning-related *rec*A1 and *end*A1 background	New England Biolabs
EcR	*E. coli* Rosetta 2 (DE3) wildtype; BL21(DE3) derivative supplying rare-codon tRNAs (*arg*U, *arg*W, *ile*X, *gly*T, *leu*W, *pro*L) via pRARE2	Novagen
MeP	*M. extorquens* PA1, *fdh1* gene source	DSMZ, Germany
MeP4	MeP*Δfdh1Δfdh2Δfdh3Δfdh4*; homologous expression host used for operon-architecture comparison	(Ryu et al. 2024)

### Plasmid construction and strains preparation

**Fig. 1:**
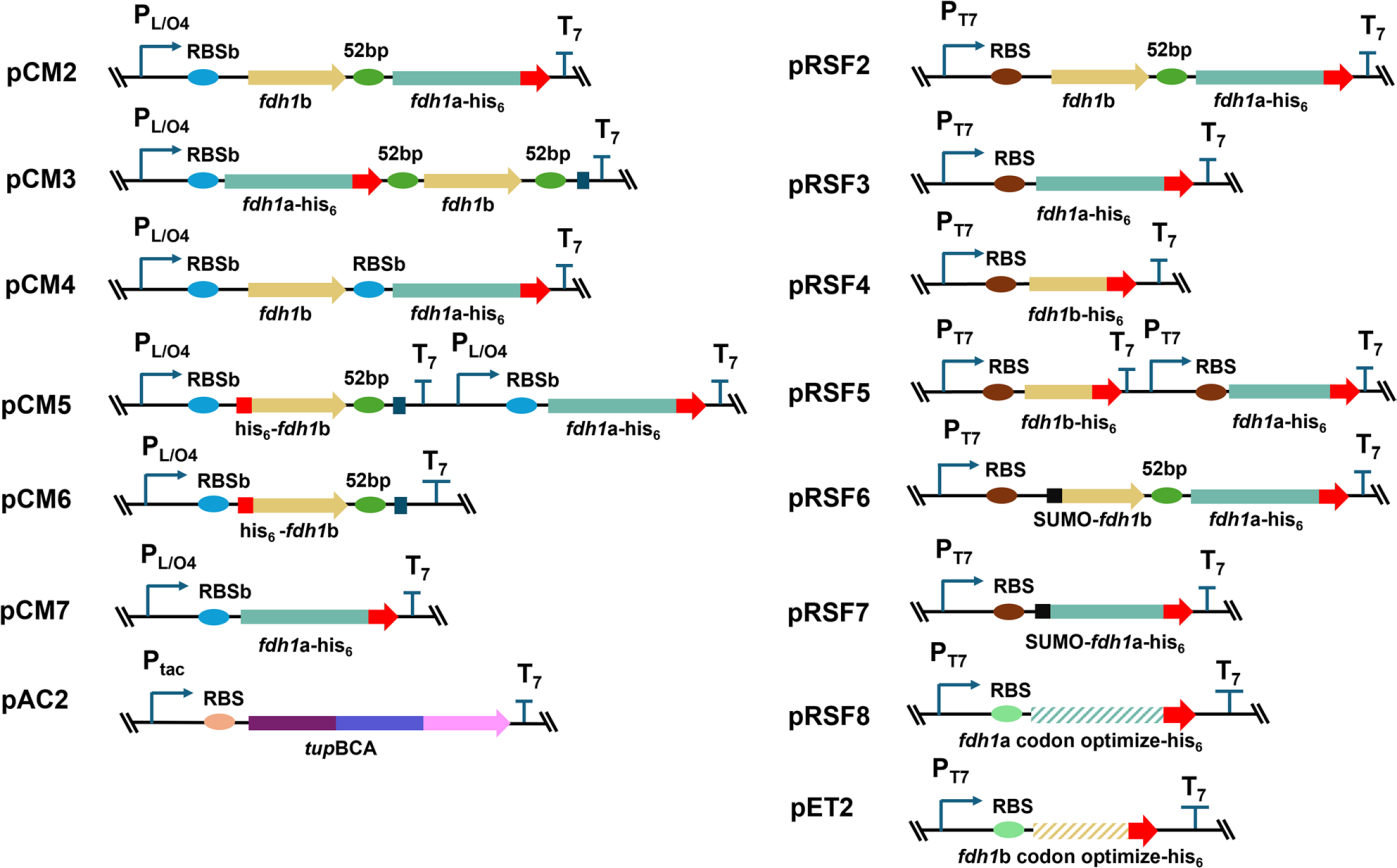
Architecture of plasmids used to express Me-FDH1

Genes encoding the Me-FDH1 α- and β-subunits (*fdh1*a and *fdh1*b) were amplified from *M. extorquens* PA1 genomic DNA and cloned under the IPTG-inducible P_L/O4_ promoter in the pBBR1-derived broad-host-range vector pCM110, generating pCM2 (Fig. 1; Table 1). The vector carries *lac*I and is compatible with *E. coli*, *M. extorquens*, and *C. necator*. Additional plasmids were constructed to evaluate the effects of gene organization, solubility tags, chaperone co-expression, and codon harmonization on heterologous expression. All plasmid backbones are summarized in Table 1, and all primers are listed in Table S1 (see Additional File).

The reference construct pCM2 contained the native *fdh1*b–*fdh1*a arrangement, including the 52 nucleotides (52-nt) intergenic region, and a C-terminal hexa-histidine tag (His_6_-tag) on the α-subunit. RNA secondary structure of the 52-nt intergenic region was predicted using RNAfold ViennaRNA package (Lorenz et al. 2011) and the model was deposited in ModelArchive ma-ny5ny (see Additional File: Figure S1). In pCM3, the gene order was reversed and the β-subunit carried an N-terminal His_6_-tag. In pCM4, the native gene order was retained, but a strong synthetic ribosome-binding site (RBS) was introduced upstream of *fdh1a*. In pCM5, the α- and β-subunits were expressed from separate promoter-RBS units. Single-subunit plasmids were also constructed: pCM6 encoded His_6_-tagged *fdh1b*, whereas pCM7 encoded His_6_-tagged *fdh1a* with a strong RBS (Fig. 1; Table 1).

Plasmids pCM4, pCM6, and pCM7 were derived from pCM2 by restriction digestion, PCR amplification of the desired fragments, and In-Fusion- or ligase-based assembly. pCM3 and pCM5 were subsequently constructed from the pCM6 backbone by insertion of PCR-amplified *fdh1a*-containing fragments and, for pCM5, an additional promoter-terminator cassette. When appropriate restriction sites were unavailable, fragments were combined by overlap PCR. All constructs were verified by restriction analysis and DNA sequencing.

For specific tungstate transport, the high-affinity *tup*BCA operon was amplified from *M. extorquens* PA1 and cloned into the tac-promoter vector pAC1, generating pAC2 (Fig. 1; Table 1). The operon corresponds to *Mext*_2850–*Mext*_2852. This construct was used to complement *E. coli* strains lacking an effective endogenous tungstate uptake route.

To examine whether general folding support improves Me-FDH1 production, the chaperone plasmids pG-KJE8 and pG-Tf2 (TaKaRa) were used. pG-KJE8 expresses *DnaK*-*Dna*J-*Grp*E together with *Gro*EL-*Gro*ES, whereas pG-Tf2 expresses *Gro*EL-*Gro*ES with trigger factor (Nishihara et al. 1998, 2000).

### Culture conditions and production of recombinant Me-FDH1

For *E. coli*, a modified M9 medium supplemented with 1 g/L yeast extract and 10 g/L glucose was used as described previously (Ryu et al. 2024, 2025). To support tungsten cofactor formation, sodium tungstate (Na_2_WO_4_, 30 µM) was added to the medium after autoclaving by filter sterilization (Ryu et al. 2025). Cultures were grown at 30 °C and 200 rpm to mid-log phase (OD_600_ ~0.4–0.8), induced with 0.5 mM IPTG, and incubated at 30 °C for 16–18 h. When chaperone plasmids were used, arabinose (0.5 mg/mL) or tetracycline (10 ng/mL) was added together with IPTG according to the plasmid system. Antibiotics were used at the following concentrations: kanamycin 50 µg/mL, ampicillin 100 µg/mL, and tetracycline 10 µg/mL. Cultivation and induction of *M. extorquens* MeP4 carrying pCM plasmids were performed as described previously (Jang et al. 2018; Ryu et al. 2024, 2025).

### Protein extraction, purification and expression analysis

Cells were harvested after induction, washed, and resuspended in lysis buffer (20 mM MOPS, 200 mM NaCl, 20 mM imidazole). Cell disruption was performed by sonication on ice, and soluble and insoluble fractions were separated by centrifugation (13000 *g*, 10–20 min, 4 °C) as described previously (Ryu et al. 2024, 2025).

His_6_-tag proteins were purified from crude extracts under nondenaturing conditions using Nickel-Nitrilotriacetic Acid (Ni-NTA) Bind Resin column (Qiagen). Eluted proteins were concentrated and buffer-exchanged with Amicon^®^ Ultra-15 centrifugal filters (30 kDa cutoff; Millipore, Darmstadt, Germany). Protein concentrations were determined by the Bradford assay. Expression levels were estimated by SDS–PAGE densitometry against bovine serum albumin (BSA) standards (Thermo Scientific) and are reported as the combined intensities of the α- and β-subunit bands when both were present.

Protein production was analyzed by SDS-PAGE and Western blotting. His_6_-tagged proteins were detected with a mouse monoclonal anti-His_6_ antibody and an alkaline phosphatase-conjugated rabbit anti-mouse secondary antibody IgG H&L (Abcam, ab97043) followed by BCIP/NBT Liquid Substrate System (Sigma-Aldrich, B1911-100ML) development.

### Enzyme activity assay

Enzyme activity was measured at 30 °C for 1 min under conditions adapted from a previous study (Jang et al. 2018). For the formate oxidation assay, reactions were performed at pH 7.0 with 30 mM sodium formate, and NADH formation was monitored at 340 nm (ε_340_ = 6220 M^− 1^ cm^− 1^). All assays were performed in triplicate. One unit (U, µmol min^− 1^) of activity was defined as the amount of enzyme that formed 1 µmol of NADH per min under the assay conditions.

### RT-PCR and metal analysis

To quantify *fdh1*a and *fdh1*b transcripts in *E. coli*, cells were grown in modified M9 medium and induced at mid-log phase. Pellets were immediately stabilized with RNAprotect™ Bacteria Reagent (Qiagen) and total RNA was isolated with the NucleoSpin RNA isolation kit (Macherey-Nagel, Germany). First-strand cDNA was synthesized with the iScript™ cDNA Synthesis Kit (Bio-Rad). Quantitative PCR was performed on a StepOne Real-Time PCR system (Applied Biosystems, USA) using SYBR Green chemistry. Transcript levels were normalized to the housekeeping gene *rpo*D (RNA polymerase sigma factor), and relative mRNA levels were calculated using the ΔΔCt method (Zhou et al. 2015). All assays were performed in duplicate, and no-template controls were included as negative controls.

Iron contents of purified protein preparations were measured by HR-ICP-MS as described previously (Ryu et al. 2024, 2025). Purified proteins were digested in nitric acid before analysis. Tungsten content of the α-subunit was measured for selected samples when sufficient material was available.

### Codon harmonization

Codon optimization was performed by codon harmonization, in which host codon usage is adjusted to approximate native translation kinetics (Schmidt et al. 2023). Codon frequencies were weighted by amino acid abundance in the complete host proteome. The abundance of amino acid A was defined by Eq. (1) (see Additional File: Figure S2):

1 $$Abundance\:\left(\boldsymbol{A}\right)=\:\frac{{n}_{\:}\left(\boldsymbol{A}\right)}{\sum\:n\left(\boldsymbol{A}\right)}\times\:100$$

where, n(***A***) is the number of occurrences of amino acid ***A***, and ∑n(***A***) is the total number of amino acids in the proteome.

Translation speed for each codon was estimated according to Eq. (2) (see Additional File: Figure S2):

2 $$Translation\:speed=Codon\:frequency\:\times\:Abundance$$

The harmonized *fdh1*a and *fdh1*b sequences for *E. coli* were deposited in NCBI GenBank under accession numbers PX353735 and PX353736, respectively. The codon-harmonized genes were cloned into pRSF-Duet-1 and pET-Duet-1 (Fig. 1) to generate pRSF8 and pET2.

### In vitro translation

To test whether the α-subunit can be synthesized in the absence of cellular proteolysis, cell-free expression was performed with the PURExpress^®^ In vitro Protein Synthesis Kit (New England Biolabs, USA) supplemented with murine RNase Inhibitor (NEB). PCR fragments containing the promoter, gene, and terminator regions were amplified from pRSF2, pRSF3, pRSF4, and pRSF5 using the primer pairs listed in Table S1 (see Additional File) and were purified before use as templates.

Each 25 µL reaction contained 10 µL Solution A, 7.5 µL Solution B, 0.5 µL RNase inhibitor, approximately 0.9–1.0 µg purified PCR template, and nuclease-free water. Reactions were incubated at 37 °C for 4 h and then cooled on ice. Products were concentrated with 10 kDa MWCO centrifugal filter (Amicon^®^ Ultra-0.5) and analyzed by SDS-PAGE. Because the PURExpress system lacks ATP-dependent proteases and most maturation pathways, it allows detection of proteins that may be unstable in vivo.

## Results and discussion

### Expression of Me-FDH1 in *Escherichia coli* K-12

**Fig. 2:**
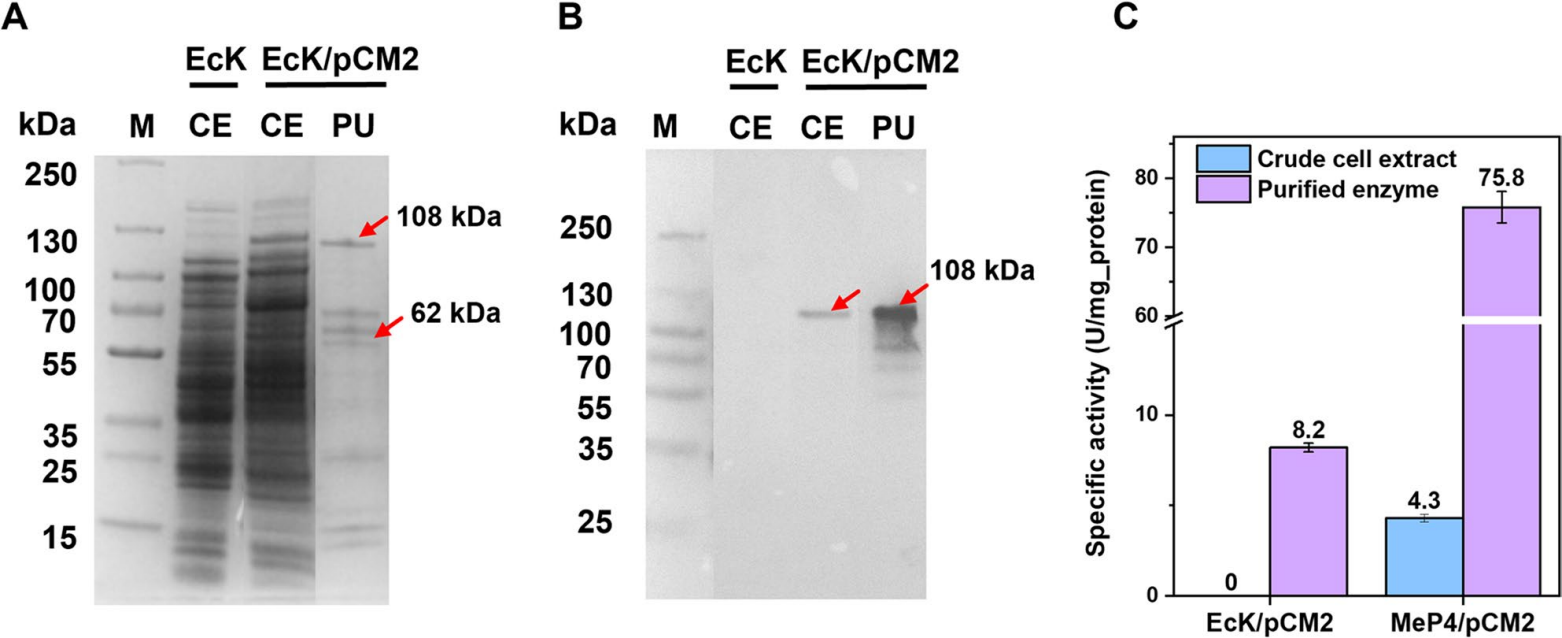
Expression and activity of Me-FDH1 in *E. coli* K-12. **A** SDS-PAGE; (**B**) Western blot; (**C**) Enzyme activity of crude cell lysate and purified enzyme. EcK and EcK/pCM2 represent *E. coli* K-12 wildtype and recombinant host harboring pCM2, respectively, MeP4/pCM2 represents recombinant host harboring pCM2. In (**A**) and (**B**); lane M indicates protein ladder, lane CE indicates crude cell extract, lane PU indicates Ni-affinity purified protein sample. Red arrows indicate the band corresponding to alpha (108 kDa) and beta (62 kDa) subunits. Enzyme activity values were measured based on formate oxidation with triplicate measurements averaged, and the error was less than 5%

Me-FDH1 was first expressed in wild-type *E. coli* K-12 MG1655 carrying pCM2, the reference plasmid described in Fig. 1; Table 1. Cultures were supplemented with sodium tungstate (30 µM) to support W-bis-MGD biosynthesis. Although SDS-PAGE of crude extracts did not reveal distinct bands at the expected positions of the α- and β-subunits, indicating very low expression in the host background (Fig. 2A), Western blot analysis using an anti-His antibody clearly detected the His_6_-tagged α-subunit in both crude extracts and Ni-NTA purified fractions (Fig. 2B). After purification, both α- and β-subunits became visible by SDS-PAGE, consistent with co-purification of the β-subunit through association with the His_6_-tagged α-subunit, as reported previously for Me-FDH1 (Ryu et al. 2024, 2025) (Fig. 2A, B; Table 2).

**Table 2:** Summary of Me-FDH1 expression in various *E. coli*

Host strain for Me-FDH1 production	Expression level (% of total soluble protein)^a^	Enzyme activity (U/mg)^b^	Molybdate transporter^c^
EcB/pCM2	~ 1	0	-
EcBL/pCM2	~ 1	0	-
EcBLP/pCM2	~ 1	0	-
EcBW/pCM2	~ 1	13.9 ± 2.1	+ (ModABC)
EcC/pCM2	~ 1	< 0.5	+ (ModABC)
EcJM/pCM2	~ 1	6.6 ± 1.9	+ (ModABC)
EcK/pCM2	~ 1	8.2 ± 0.5	+ (ModABC)
EcKT/pCM2	~ 1	4 ± 0.8	+ (ModABC)
EcMC/pCM2	~ 1	< 0.5	+ (ModABC)
EcWA/pCM2	~ 1	4.1 ± 0.7	+ (ModABC)
EcW/pCM2	~ 1	3.3 ± 0.3	+ (ModABC)
EcXL/pCM2	~ 1	< 0.5	-
EcR/pCM2	~ 1	< 0.5	-
EcBL/pRSF2/pAC2^d^	~ 1	2.6 ± 0.6	++ (TupBCA)

^*a*^Expression level was estimated by SDS-PAGE densitometry of the soluble fraction obtained after cell disruption and centrifugation, and is reported as the percentage of total soluble protein. Values below the detection limit are indicated as < 1%

^*b*^Estimated from enzyme assay for formate oxidation reaction with purified samples

^*c*^Molybdate transporter system (*mod*ABC) is non-specific to molybdate and possible to bind tungstate (Otrelo-Cardoso et al. 2017)

^*d*^The TupBCA transporter from *M. extorquens* PA1 was expressed for tungstate uptake (Table 1)

Formate oxidation activity was not detectable in the crude cell extract, but the purified enzyme showed a specific activity of 8.2 ± 0.5 U mg^− 1^ (Fig. 2C; Table 2). Because Me-FDH1 activity requires proper insertion of the tungsten-containing cofactor (W-bis-MGD), this result indicates that *E. coli* can produce catalytically competent Me-FDH1. However, the activity remained far below that of the same enzyme produced in the homologous *M. extorquens* system, where purified recombinant Me-FDH1 reached 84.5 U mg^− 1^ under comparable expression conditions (Table 3), consistent with previous reports of approximately 80–100 U mg^− 1^ for the native/homologous enzyme (Park et al. 2024; Ryu et al. 2024).

**Table 3:** Quantification of Me-FDH1 during Ni-NTA affinity purification, activity of purified enzymes, and Fe content of selected proteins

Strain	Plasmid	Cell mass (mg)	Protein in crude cell extract (mg)	FDH1 proteins after Ni-NTA			Specific production (mg/g CDW)^b^	Specific activity (U/mg protein)^b^	Fe content (mol/mol)
				Quantity (mg)	Purity (%)^a^				
EcK	pCM2	1380	711.01	4.69	22	0.7		8.0	17.6
	pCM3	1560	1044.08	4.03	5.0	< 0.1		0.3	14.3
	pCM4	1680	1127.33	3.88	0.8	< 0.1		0.2	12.9
	pCM5	1680	1630.42	10.23	72	6.4		1.3	5.7
	pCM6	1650	1633.63	22.91	99	8.7		n/a	3.2
	pCM7	990	578.56	2.21	0.5	< 0.1		n/a	n/a
MeP4	pCM2	1215	439.76	4.38	99	3.6		84.5	18.2
	pCM3	1097	199.41	1.07	99	1.0		78.7	16.5
	pCM4	1207	527.24	1.33	99	1.1		63.7	19.6
	pCM5	1284	451.52	6.61	99	5.1		65.9	22.4
	pCM6	1359	559.68	2.54	80	1.5		n/a	2.5
	pCM7	1174	368.69	0.74	9	< 0.1		n/a	n/a

^a^Determined by densitometry on SDS-PAGE

^b^Adjusted for purity

### Importance of molybdate/tungstate transport in *E. coli* strains

Because the K-12 result suggested that *E. coli* can synthesize and insert W-bis-MGD, we next examined Me-FDH1 production across a broader panel of *E. coli* strains listed in Table 1 and summarized in Table 2.

The strain panel was selected to probe four distinct physiological and engineering features that could limit Me-FDH1 production. T7-based high-expression strains, namely BL21 (DE3), BL21 Star (DE3) pLysS, and JM109 (DE3), were used to evaluate transcriptional and translational capacity; JM109 (DE3) has previously supported functional heterologous expression of several formate dehydrogenases (Alissandratos et al. 2014). Rosetta 2 (DE3) supplies rare tRNAs for codons that are infrequent in *E. coli* (Lipinszki et al. 2018). K-12 Shuffle T7 provides a redox-engineered cytoplasm compatible with disulfide-bond formation (Lobstein et al. 2012). K-12-derived and natural-isolate strains (MG1655, BW25113, W3110, W, B, C, XL-1 Blue, MC1061) span different ModABC uptake statuses and physiological backgrounds, and MC1061 has been used previously for heterologous expression of FDH cofactor-insertion chaperones (Böhmer et al. 2014). This design allowed us to separate the contribution of metal uptake from the contribution of the host expression machinery.

As in K-12, none of the strains showed clearly visible Me-FDH1 bands in crude lysates by SDS-PAGE, whereas Western blot analysis detected the α-subunit broadly across recombinant strains (see Additional File: Figure S3). After Ni-affinity purification, active Me-FDH1 was recovered only from a subset of strains, with purified-enzyme specific activities ranging from 3.3 to 13.9 U mg^− 1^ (Table 2). Among these, BW25113 gave the highest activity, followed by K-12 MG1655, JM109, WA, and W3110 (Table 2).

A notable common feature of the active strains was the presence of a functional *mod*ABC molybdate transporter system (Table 2). The *Mod*ABC transporter is known to transport tungstate as well as molybdate, albeit with lower specificity than dedicated tungstate transporters (Leimkühler et al. 2011; Otrelo-Cardoso et al. 2017). In contrast, strains lacking a functional ModABC system showed no detectable Me-FDH1 activity (Table 2). One exception was *E. coli* C, which carries *mod*ABC but still showed little or no activity, indicating that transporter availability is necessary but not sufficient. Because all tested *E. coli* strains inherently possess the molybdopterin biosynthetic machinery (see Additional File: Figure S3–S4; Table S2), these results suggest that tungsten uptake, rather than the absence of the cofactor-biosynthetic pathway itself, is the primary requirement for active Me-FDH1 formation in *E. coli*.

To test this interpretation directly, the high-affinity tungstate transporter *Tup*BCA from *M. extorquens* was introduced on plasmid pAC2 (Fig. 1; Table 1) into *E. coli* BL21 (DE3), which lacks a functional *Mod*ABC system and did not produce active Me-FDH1 from pCM2 alone (Table 2). In both EcBL/pCM2/pAC2 and EcBL/pRSF2/pAC2, the α-subunit became detectable after purification, and the purified enzyme displayed activity of approximately 2.6 U mg^− 1^ (Table 2; see Additional File: Figure S5). Thus, either native *Mod*ABC or heterologous *Tup*BCA can provide the metal-uptake route required for Me-FDH1 maturation in *E. coli*. At the same time, the *Tup*BCA-complemented strains still produced only low amounts of weakly active enzyme, indicating that tungsten uptake is necessary for activity but is not the dominant bottleneck for production yield.

While tungsten uptake is required for Me-FDH1 maturation, the present data indicate that it is not the dominant limiting factor under the conditions tested. Increasing tungstate supplementation and the expression of TupBCA did not result in proportional increases in enzymatic activity. Furthermore, constructs that differ substantially in α-subunit accumulation show comparable specific activity per unit of recovered protein (Table 2). Together, these observations indicate that the primary limitation arises upstream of cofactor insertion, most likely at the level of α-subunit maturation. Quantitative analysis of intracellular tungsten incorporation (for example by ICP-MS) will be required to refine this conclusion and is an important direction for future work.

### Effect of gene organization on expression of Me-FDH1

**Fig. 3:**
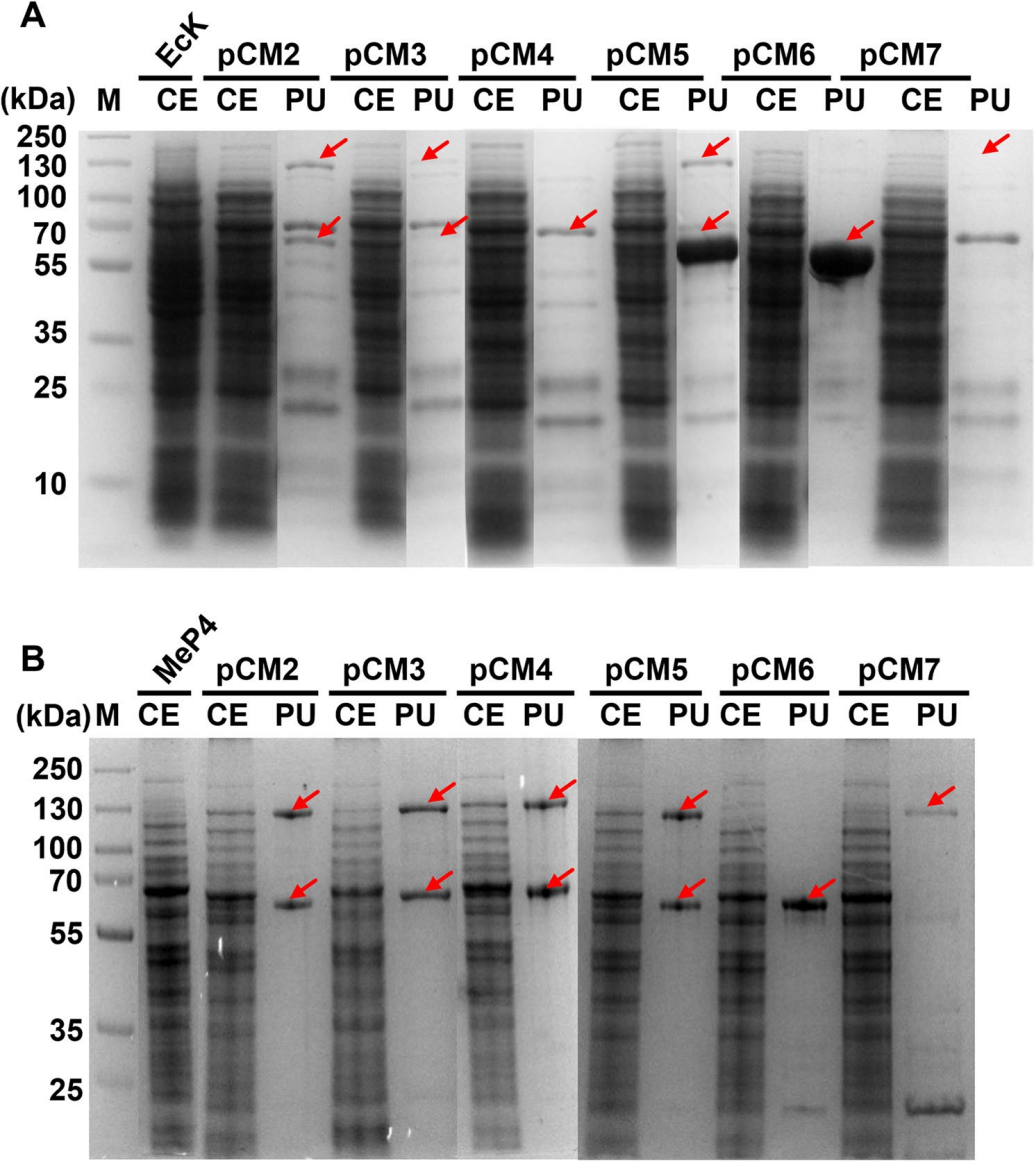
Effect of gene organization for heterologous expression of Me-FDH1. **A** SDS-PAGE analysis shows protein expression in EcK strain; (**B**) SDS-PAGE analysis shows protein expression in MeP4 strain. Lane M, protein ladder; Lane CE, crude cell extract; Lane PU, purified protein. Red arrows indicate the band corresponding to alpha (108 kDa) and beta (62 kDa) subunits

The preceding experiments indicated two major problems when Me-FDH1 is produced in *E. coli*: (i) low enzyme yield and (ii) low specific activity. To determine whether these problems arise at the transcriptional or post-transcriptional level, we constructed a series of plasmids with altered operon architectures (Figs. 1 and 3; Table 1). The reference construct pCM2 contains the native *fdh1*b–*fdh1*a arrangement separated by a 52-nt intergenic region. This region was predicted to form an extensive secondary structure (see Additional File: Figure S1; ModelArchive: ma-ny5ny), which could in principle influence transcript stability or translation efficiency through a riboswitch-like mechanism (Serganov and Nudler 2013). Accordingly, pCM3, pCM4, and pCM5 were designed to alter gene order, translation signals, or operon structure, whereas pCM6 and pCM7 expressed the β- and α-subunits individually (Fig. 1; Table 1).

**Fig. 4:**
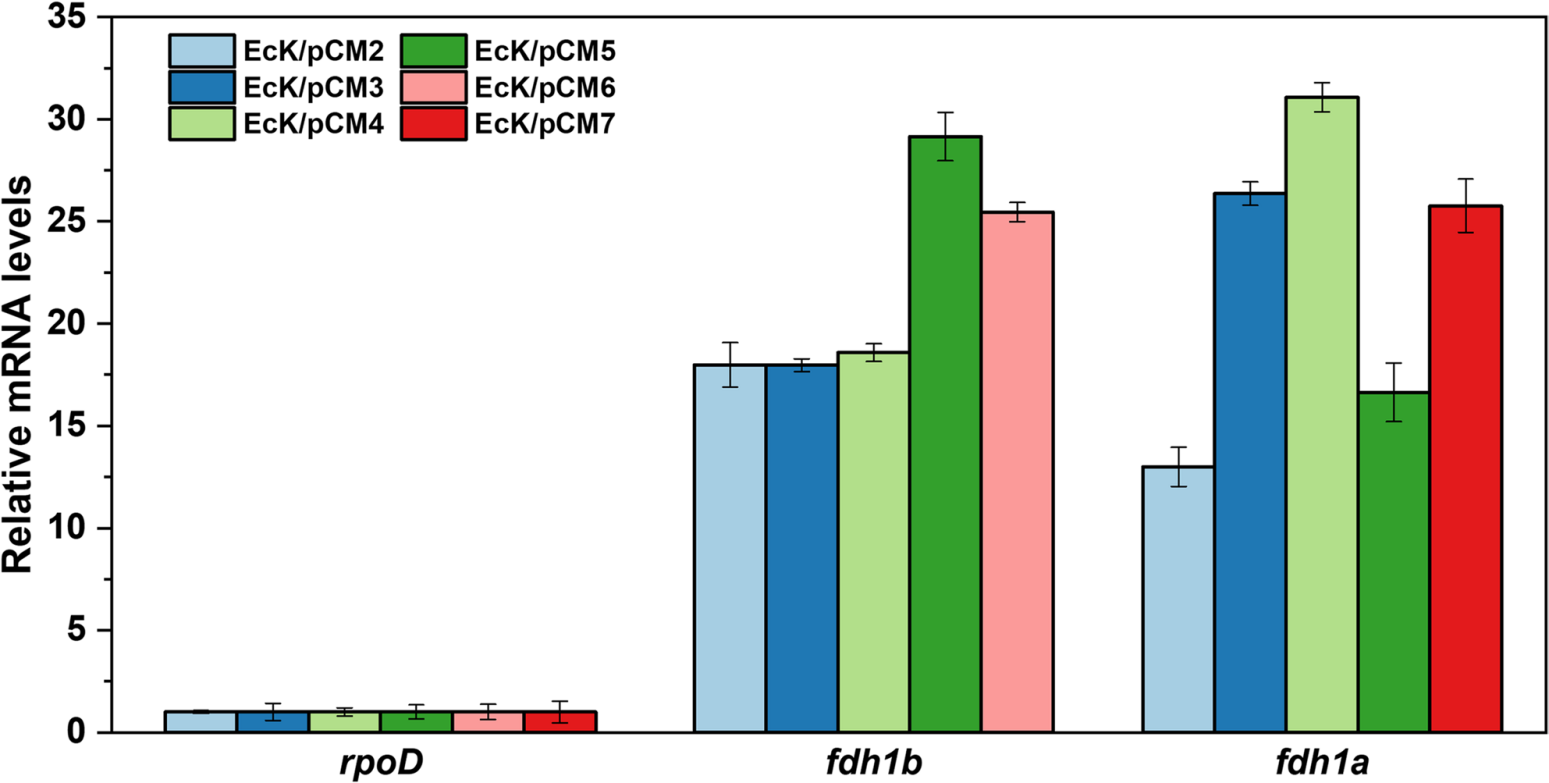
Analysis of mRNA synthesis from various gene organizations for heterologous expression of Me-FDH1 by RT-PCR. Relative mRNA levels were quantified by RT-PCR and normalized against the housekeeping gene *rpo*D as a control. The relative mRNA levels were calculated as the average value from triplicate measurements, with the error in each measurement being less than 5%

RT-PCR analysis showed that all constructs produced full-length transcripts at comparable levels (Fig. 4). Quantitative RT-PCR was normalized to *rpo*D, using the ΔΔCt approach described previously (Zhou et al. 2015). This result indicates that transcription of *fdh1*a and *fdh1*b is not the primary bottleneck and that the native 52-nt intergenic region does not cause severe premature transcription termination under the tested conditions. The observed invariance of full-length transcript levels across pCM2 to pCM5 (Fig. 4) and the efficient translation of these constructs in the PURExpress cell-free system (see Section “In vitro translation”) together indicate that mRNA secondary structure or translational coupling, although predicted by RNAfold, does not impose a dominant in vivo constraint. Direct structural probing by SHAPE (Selective 2’-Hydroxyl Acylation analyzed by Primer Extension) or DMS-MaPseq (Dimethyl Sulfate Mutational Profiling with sequencing) was therefore not pursued. Although ribosome profiling was not performed, the efficient translation of both subunits in PURExpress, combined with comparable mRNA levels across constructs (Fig. 4), provides direct evidence that translation initiation and elongation are not rate-limiting for Me-FDH1 production in *E. coli*.

In contrast, protein recovery and enzyme performance were strongly affected by plasmid configuration (Fig. 3A; Table 3). In *E. coli* K-12, pCM2 gave the best balance of α-subunit accumulation and enzyme activity, yielding 0.7 mg g^− 1^ CDW purified Me-FDH1 with a specific activity of 8.0 U mg^− 1^ (Table 3). Reversing gene order (pCM3) or introducing a stronger downstream RBS (pCM4) sharply reduced α-subunit recovery and lowered activity to 0.3 and 0.2 U mg^− 1^, respectively (Table 3). By contrast, pCM5, in which the two genes were separated into individual operons, gave a large increase in recovered protein because the β-subunit accumulated efficiently, but the specific activity remained low (1.3 U mg^− 1^), indicating that α-subunit production remained limiting (Table 3). This interpretation is reinforced by the single-subunit constructs: pCM6 yielded a large amount of purified β-subunit (8.7 mg g^− 1^ CDW), whereas pCM7 yielded almost no purified α-subunit (< 0.1 mg g^− 1^ CDW) (Table 3).

A parallel experiment in the homologous host *M. extorquens* MeP4 led to the same qualitative conclusion regarding subunit asymmetry (Fig. 3B; Table 3). When both subunits were co-expressed, pCM2, pCM3, pCM4, and pCM5 all yielded highly active enzyme preparations (63.7–84.5 U mg^− 1^), but expression of the α-subunit alone from pCM7 again resulted in extremely poor recovery (< 0.1 mg g^− 1^ CDW) (Table 3). By contrast, the β-subunit alone was readily produced from pCM6 in both hosts (Fig. 3A, B; Table 3). Together, these data indicate that the β-subunit is not merely a passive partner in the final αβ complex, but likely contributes to productive α-subunit accumulation, stabilization, and/or maturation.

The Fe-content analysis supports this interpretation but should be viewed as construct-dependent rather than as a general defect in all *E. coli* preparations (Table 3). The theoretical Fe content of Me-FDH1 holoenzyme is 20 mol/mol protein, with 14 Fe atoms assigned to the α-subunit and 6 to the β-subunit (Yoshikawa et al. 2022). The co-expressed holoenzyme from EcK/pCM2 contained 17.6 mol Fe/mol protein, close to the value observed for MeP4/pCM2 (18.2 mol/mol), indicating that near-complete Fe incorporation is possible in *E. coli* when productive co-expression occurs (Table 3). In contrast, constructs enriched in the β-subunit and poor in the α-subunit, especially pCM5 and pCM6, showed much lower Fe contents in both hosts (Table 3). Notably, β-subunit-only preparations from EcK/pCM6 and MeP4/pCM6 contained only about half of the expected Fe for the β-subunit alone, suggesting incomplete Fe-S cluster incorporation or reduced cluster stability in the absence of coordinated holoenzyme assembly. Thus, the dominant bottleneck is α-subunit accumulation, whereas impaired Fe-S maturation appears to be most evident in constructs where α-subunit co-production is compromised.

The parsimonious interpretation of these data is that the β-subunit stabilizes the α-subunit through post-translational shielding. The α-subunit is translated efficiently in the absence of the β-subunit in the PURExpress cell-free experiment (see Section “In vitro translation”), and is transcribed normally in pCM7, yet does not accumulate in vivo. The β-subunit therefore appears to protect a partially folded α-subunit from quality control recognition, an arrangement that parallels the role of the FdsC/FdsD chaperones in the assembly of the *R. capsulatus* FDH α-subunit with bis-MGD (Böhmer et al. 2014; Hartmann and Leimkühler 2013).

A decisive test of the timing of β-subunit action will require orthogonal induction of the two subunits, for example by placing β under an arabinose-inducible promoter and α under an IPTG-inducible promoter. If the β-subunit acts co-translationally, simultaneous induction should be necessary for α-subunit accumulation; if it acts by post-translational shielding, prior induction of the β-subunit should be sufficient.

### SUMO fusion tag and general chaperones did not improve α-subunit in *E. coli*

**Fig. 5:**
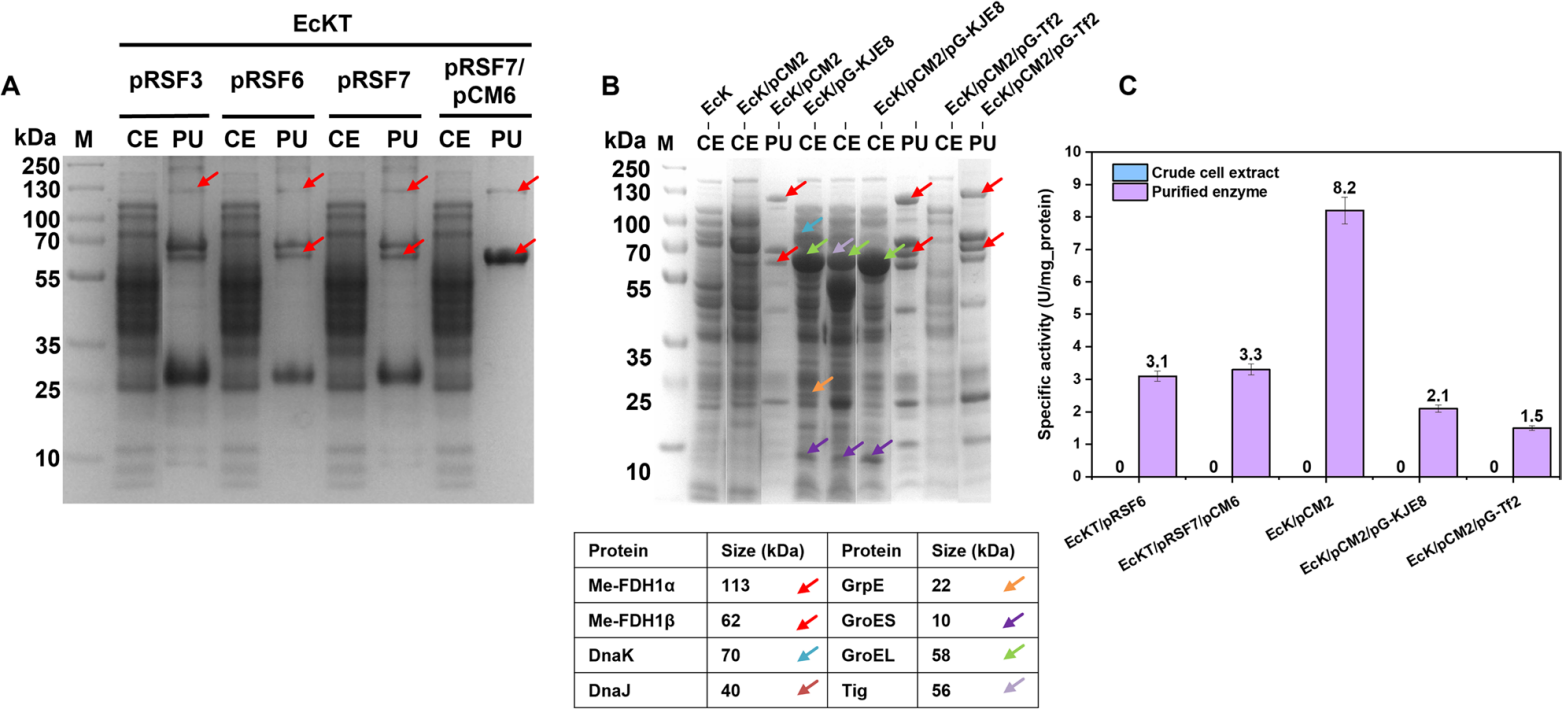
Effect of SUMO tag and co-expression of chaperones on Me-FDH1 expression in *E. coli.* **A** SDS-PAGE analysis of the effect of SUMO-tagged Me-FDH1 in *E. coli* K-12 Shuffle^®^ T7; (**B**) SDS-PAGE analysis of the effect of chaperone co-expression in *E. coli* K-12 MG1655; (**C**) Enzyme activity assay for formate oxidation. Lane M, protein ladder; Lane CE, crude cell extract; Lane PU, purified protein. Red arrows indicate the band corresponding to alpha (108 kDa) and beta (62 kDa) subunits. Enzyme activity values were measured based on formate oxidation with triplicate measurements averaged, and the error was less than 5%

Because the α-subunit accumulated poorly in *E. coli*, we next tested whether solubility enhancement or generic folding support could improve its production. SUMO-based constructs were designed using the plasmids shown in Fig. 1; Table 1 and expressed in *E. coli* K-12 Shuffle^®^ T7 (EcKT), a T7-compatible strain that retains the native *Mod*ABC uptake system and therefore minimizes possible confounding effects from tungsten limitation (Table 1; Fig. 5A). None of the tested SUMO configurations increased α-subunit accumulation detectably (Fig. 5A). Even in the best SUMO-related cases, purified preparations showed only low activities of approximately 3.1–4.0 U mg^− 1^ (Fig. 5C), which remained below the activity of the original EcK/pCM2 enzyme (Tables 2 and 3). Thus, improved solubility via a SUMO fusion was insufficient to overcome the main production barrier.

We then examined whether co-expression of molecular chaperones could improve α-subunit folding. Chaperone systems encoded on pG-KJE8 and pG-Tf2 (Table 1) provide GroEL-GroES together with either DnaK-DnaJ-GrpE or trigger factor, respectively (Nishihara et al. 1998, 2000). Although the chaperones themselves were strongly expressed, no improvement in α-subunit recovery was observed. SDS-PAGE still showed only a faint α-subunit band after purification (Fig. 5B), and Me-FDH1 activity did not increase beyond the level of the original construct (Fig. 5C). These results indicate that the limitation in α-subunit production is not rescued by a generic solubility tag or by broad cytosolic chaperone overexpression.

### Codon harmonization did not improve α-subunit production in *E. coli*

Because transcription was not limiting (Fig. 4) and neither SUMO fusion nor general chaperones improved α-subunit accumulation (Fig. 5), we next investigated whether inefficient co-translational folding due to host-specific translation kinetics could be responsible. Translation-rate mismatch is known to affect protein folding and stability in heterologous hosts (Francis and Page 2010). Therefore, the *fdh1*a and *fdh1*b genes were redesigned by codon harmonization to better mimic the native translational speed landscape of *M. extorquens* in *E. coli* (Schmidt et al. 2023).

Comparison of codon usage among *M. extorquens*, *C. necator*, and *E. coli* showed substantial differences between *M. extorquens* and *E. coli*, particularly in the α-subunit gene, whereas *M. extorquens* and *C. necator* were more similar (see Additional File: Figure S6). After harmonization, the GC contents of the optimized genes decreased markedly, and the predicted correlations between native and host translation speeds improved for both subunits (see Additional File: Figure S6). The optimized sequences also retained only 77.91% and 75.80% nucleotide identity for the α- and β-subunit genes, respectively (see Additional File: Figure S6A, B). The codon-optimized genes were cloned into the T7-based vectors shown in Fig. 1; Table 1.

**Fig. 6:**
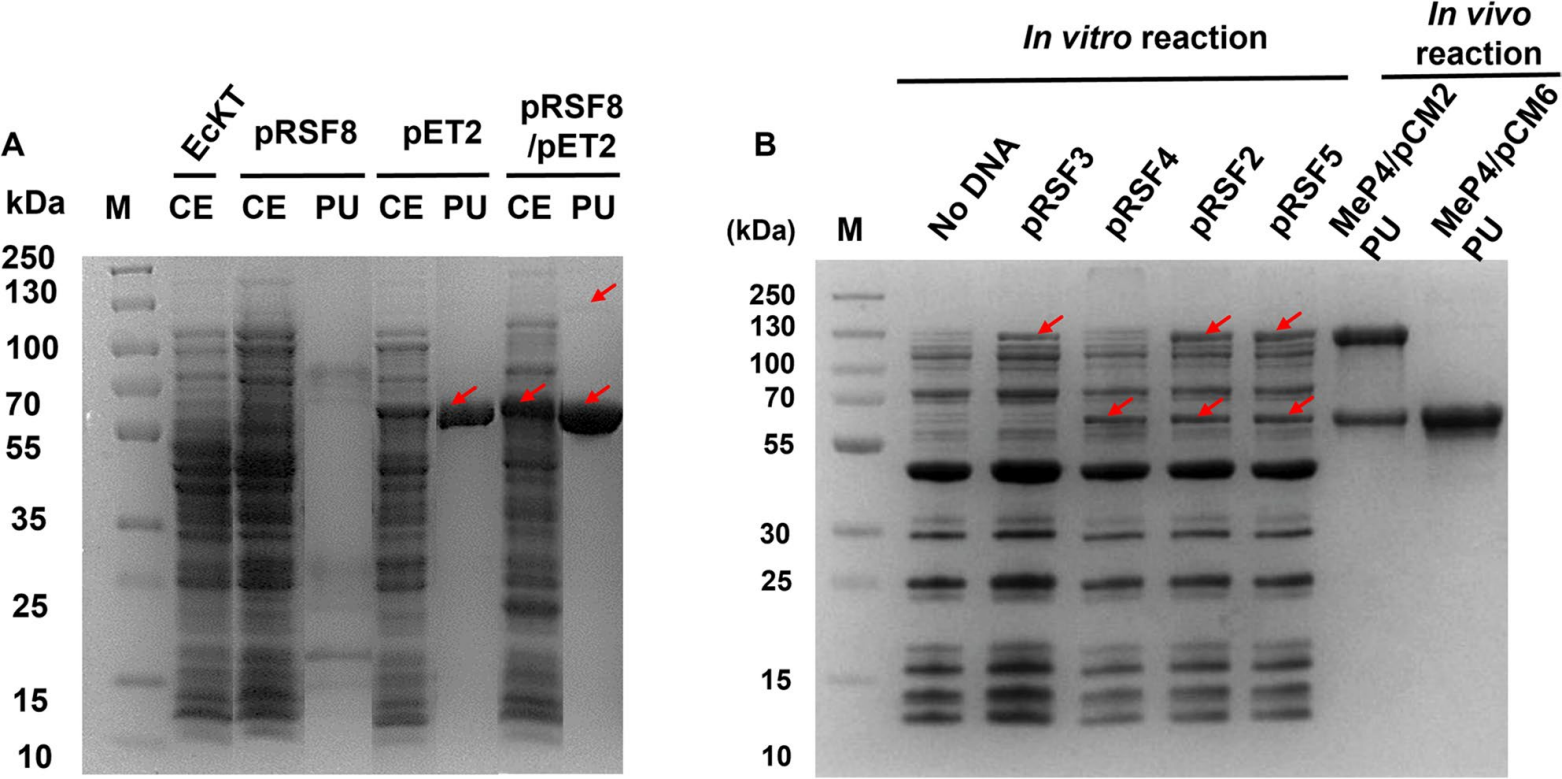
Codon optimization and In vitro translation of Me-FDH1. **A** SDS-PAGE analysis of codon-optimized *fdh1* expression in *E. coli* K-12 Shuffle^®^ T7; (**B**) SDS-PAGE analysis of Me-FDH1 produced in an in vitro expression reaction. Lane M, protein ladder; Lane CE, crude cell extract; Lane PU, purified protein. Red arrows indicate the band corresponding to alpha (108 kDa) and beta (62 kDa) subunits

Despite these substantial sequence changes, codon harmonization did not improve α-subunit accumulation. Neither EcKT/pRSF8 nor EcKT/pRSF8/pET2 showed increased α-subunit recovery relative to the corresponding non-optimized controls, and the purified proteins remained only weakly active (Fig. 6A). When expressed individually, neither subunit produced detectable FDH activity, and when both were present the purified preparations still showed only low specific activities (2.8–3.9 U mg^− 1^; data not shown). Codon harmonization is effective when translation-rate mismatches perturb co-translational folding. Our data indicate that the limiting step is downstream of translation. The α-subunit is translated efficiently in the PURExpress cell-free experiment (see Section “In vitro translation”), and the harmonized gene does not accumulate better in vivo. For a large multidomain metalloenzyme such as the Me-FDH1 α-subunit, the rate-limiting steps are domain packing, Fe-S cluster incorporation, and W-bis-MGD insertion, all of which occur after the polypeptide leaves the ribosome. Codon-level tuning therefore cannot by itself overcome this limitation.

### In vitro *translation supports post-translation loss of the α-subunit *in vivo

To distinguish between failure of α-subunit synthesis and loss of the α-subunit after synthesis, we used a PURExpress cell-free transcription/translation system, which lacks ATP-dependent proteases and most cellular folding and maturation pathways (Fig. 6B). Templates derived from the constructs listed in Table 1 were used to express both subunits together (pRSF2 and pRSF5) or individually (pRSF3 and pRSF4). In this cell-free system, both α- and β-subunits were produced efficiently, and the α-subunit was clearly visible even when expressed alone (Fig. 6B). These results demonstrate that the *fdh1*a transcript is fully translatable and that ribosomes can synthesize the large α-polypeptide.

Because the PURExpress system lacks tungsten cofactor biosynthesis and Fe-S cluster assembly machinery, the proteins formed in vitro were necessarily apo-proteins. However, their successful synthesis shows that neither transcription nor the basic translational machinery is intrinsically limiting for Me-FDH1 production. A similar accumulation of apo-Me-FDH1 in the absence of tungsten has also been observed previously in *M. extorquens* and *C. necator* (Ryu et al. 2024), indicating that cofactor absence does not by itself prevent full-length polypeptide synthesis. The activity gap between *E. coli* and *M. extorquens* is therefore attributable to two coupled effects rather than a single cause. Cofactor insertion itself is not the primary limitation, as indicated by the stable specific activity across constructs and the lack of proportional response to tungstate supplementation. Under the most productive co-expression condition (EcK/pCM2), Fe content reaches 17.6 mol per mol protein, close to the theoretical 20 mol per mol holoenzyme and comparable to the native host (MeP4/pCM2, 18.2 mol/mol; Table 3), indicating efficient Fe-S assembly in this regime. The reduced activity primarily reflects the small pool of α-subunit that escapes proteolysis, combined with residual structural imperfections in that pool. Fe-S cluster assembly is impaired only when α- and β-subunits are not co-produced, consistent with a requirement for coordinated holoenzyme assembly.

Two mechanisms could in principle explain the post-translational loss of the α-subunit: proteolytic degradation and misfolding-driven aggregation. Three observations are more consistent with a proteolytic mechanism. The insoluble fraction did not contain a detectable α-subunit band, the protein was produced intact in the protease-free PURExpress system, and co-expression of the β-subunit stabilized the α-subunit even though the β-subunit is not expected to participate directly in α-subunit folding. A contribution from misfolding cannot be excluded and would be consistent with recognition of an immature apo-form by the cellular quality control network, as discussed below.

The inability of *E. coli* to functionally produce Me-FDH1 has been reported previously by Park et al. (Park et al. 2024), who could not obtain active enzyme in *E. coli* and moved to the native *M. extorquens* host for their structural study. The present study quantifies this limitation and localizes it to post-translational α-subunit loss. The heterologous-to-native activity ratio observed here (approximately 5–15%) is comparable to that reported for other complex metalloenzymes produced in *E. coli*, including W-dependent FDHs (Maia et al. 2017; Niks and Hille 2019), Mo-dependent FDHs (Böhmer et al. 2014; Hartmann and Leimkühler 2013), and [FeFe]-hydrogenases (Girbal et al. 2005; Böck et al. 2006; Kuchenreuther et al. 2010; Winkler et al. 2013). This work therefore advances the field less by improving absolute activity and more by identifying the rate-limiting step, which defines a concrete engineering target for subsequent studies.

Taken together, the data are consistent with a model in which the α-subunit is translated efficiently but remains in an immature apo-state in *E. coli* long enough to be recognized and degraded by ATP-dependent proteases of the host protein quality control network. The β-subunit reduces this window by stabilizing the α-subunit after translation. This maturation-coupled model, rather than a transcriptional or translational defect, is the parsimonious explanation for the observed bottleneck.

A direct test of the proteolysis hypothesis will require cell-free expression supplemented with purified ATP-dependent proteases, ideally Lon and ClpXP, together with reconstitution of the complex cofactor environment of Me-FDH1 (W-bis-MGD, FMN, and multiple Fe-S clusters). W-bis-MGD and intact Fe-S clusters are not available as commercial reagents, and their in vitro supply requires dedicated maturation proteins and strictly anaerobic handling (Böhmer et al. 2014; Kuchenreuther et al. 2010). Such experiments, together with tests in protease-deficient *E. coli* strains, are the most immediate next step following from this work.

## Conclusions

Although this study did not achieve high-level heterologous production of Me-FDH1 in *E. coli*, it identifies the dominant obstacle with much greater clarity. *E. coli* produced catalytically competent Me-FDH1 when tungstate uptake was supported by a functional *Mod*ABC system or by heterologous expression of *Tup*BCA (Fig. 2; Table 2), showing that the host is not fundamentally incompatible with W-bis-MGD-dependent biogenesis. The major limitation instead lies in poor accumulation and incomplete maturation of the large α-subunit. Gene-organization experiments showed that the β-subunit is readily produced on its own, whereas the α-subunit is not efficiently recovered even in the native host unless the β-subunit is present, indicating that the β-subunit contributes to productive α-subunit stabilization and/or maturation (Figs. 3 and 4; Table 3). SUMO fusion, generic chaperone co-expression, and codon harmonization did not rescue this defect (Figs. 5 and 6A), whereas cell-free translation readily produced the α-subunit in the absence of ATP-dependent proteases (Fig. 6B), strongly supporting a model in which the α-subunit is synthesized in *E. coli* but is rapidly lost through folding-coupled instability and post-translational degradation. From a practical standpoint, *C. necator* remains the more suitable heterologous host for Me-FDH1 production at present (Ryu et al. 2024, 2025).

Two non-exclusive causes may underlie the prolonged immature state of the α-subunit. The maturation-related factors of *E. coli*, including components of the W-bis-MGD pathway and the Fe-S assembly machinery, may be insufficient or mistimed relative to the overexpressed α- and β-subunits. Alternatively, these factors may be intrinsically unable to process the heterologous Me-FDH1 α-subunit efficiently. Sequence identity between the *E. coli* and *M. extorquens* machineries is low for several pathway steps (Table S2, see Additional File), and in *R. capsulatus* FDH, the dedicated maturation chaperones FdsC and FdsD are required for activity but have no close *E. coli* homolog (Böhmer et al. 2014; Hartmann and Leimkühler 2013). Both mechanisms are consistent with the observed phenotype and motivate the engineering strategies outlined below.

Four concrete engineering strategies are suggested by the identified bottleneck. The first is co-expression of *M. extorquens* maturation-relevant factors, because protein sequence identity of several steps in the W-bis-MGD pathway is low between *M. extorquens* and *E. coli* (Table S2, see Additional File). The second is comparative transcriptomic and proteomic profiling of *E. coli* and *M. extorquens* under identical cultivation conditions, to identify W-bis-MGD biosynthesis and Fe-S cluster assembly genes that are expressed at insufficient levels in *E. coli* relative to *M. extorquens*; any such genes can then be targeted for controlled upregulation to reproduce the native expression balance. The third is fragment-based dissection of the α-subunit. The α-subunit contains multiple structural domains involved in Fe-S cluster binding and W-bis-MGD coordination, and the domain-level pattern of accumulation across individually expressed fragments can localize the region that is most susceptible to proteolysis, which can then guide rational stabilization. The fourth is controlled reduction of α-subunit expression rate, using weaker promoters or lower inducer concentrations, to balance synthesis against the available maturation capacity. These strategies are not mutually exclusive and can be combined.

More broadly, however, this study defines a clear engineering roadmap for adapting *E. coli* to produce Me-FDH1 and related tungsten enzymes: improve α-subunit stabilization, identify and suppress the proteolytic pathway(s) responsible for α-subunit loss, and reconstruct the folding and maturation environment required for efficient holoenzyme assembly. By pinpointing the dominant bottleneck rather than merely documenting low expression, this work advances the development of robust microbial platforms for sustainable CO_2_-to-formate biocatalysis.

## Supplementary Information

Additional file 1: Word file containing Figures S1–S6, Tables S1–2, and supplementary text supporting the results of this study.

## Data Availability

All data generated or analyzed during this study are included in this published article and its supplementary information files. The predicted structural models generated in this study have been deposited in ModelArchive under accession numbers ma-ny5ny, ma-covi8, ma-wmubt, ma-b9uc9, ma-jp91h, and ma-ifuk9. The codon-harmonized *fdh1a* and *fdh1b* sequences have been deposited in NCBI GenBank under accession numbers PX353735 and PX353736, respectively.

## Abbreviations

ATP: Adenosine triphosphate
BSA: Bovine serum albumin
CDW: Cell dry weight
FDH: Formate dehydrogenase
Fe-S cluster: Iron-sulfur cluster
[FeFe]-hydrogenases: Unique 6-iron active site [4Fe–4Fe] cluster linked to a [2Fe] subcluster
His_6_ tag: hexa-histidine tag
HR-ICP-MS: High resolution inductively coupled plasma mass spectrometry
IPTG: Isopropyl-β-D-thiogalactopyranoside
Me-FDH1: Formate dehydrogenase I from Methylorubrum extorquens
ModABC: High-affinity molybdate/tungstate ABC-type transporter system
Ni-NTA: Nickel-Nitrilotriacetic Acid
RBS: Ribosome-binding site
RPM: Revolutions per minute
RT-PCR: Reverse transcription polymerase chain reaction
SDS-PAGE: Sodium dodecyl sulfate-polyacrylamide gel electrophoresis
SUMO: Small ubiquitin-like modifier
TupBCA: High-affinity tungstate ABC-type transporter system
W-bis-MGD: Tungsto-bis(molybdopterin guanine dinucleotide) cofactor
