# Supplementary material for: Heterologous production of tungsten-dependent formate dehydrogenase I from Methylorubrum extorquens in Escherichia coli reveals α-subunit maturation as the major bottleneck
Source: Bioresour Bioprocess. 2026 Jul 24;13(1):105. doi: 10.1186/s40643-026-01092-7 (PMC13396061; doi:10.1186/s40643-026-01092-7)
Supplement: Supplementary file 1 — Additional file 1: Word file containing Figures S1–S6, Tables S1–2, and supplementary text supporting the results of this study. [file 40643_2026_1092_MOESM1_ESM.zip › Additional File.docx]

**Supplementary materials**

**Heterologous production of tungsten-dependent formate dehydrogenase I from *Methylorubrum extorquens* in *Escherichia coli* reveals α-subunit maturation as the major bottleneck**

# Supplementary figures

*
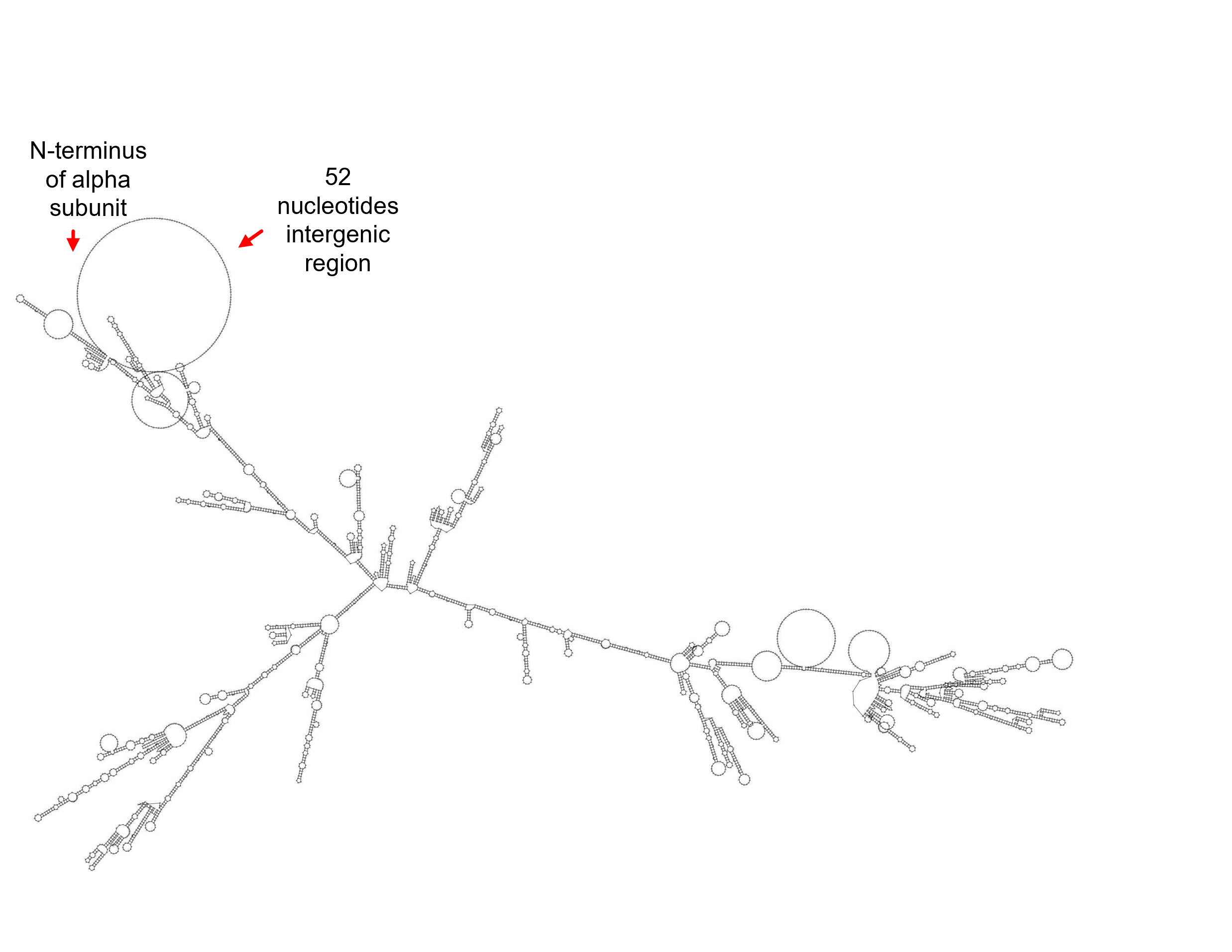
*

**Figure S1. Predicted secondary structure of the 52-nucleotide intergenic region between the α- and β-subunit genes of fdh1.** Red arrows indicate the start codon of the α-subunit gene and the N-terminus (5′ end) of the 52-nucleotide intergenic region.

*
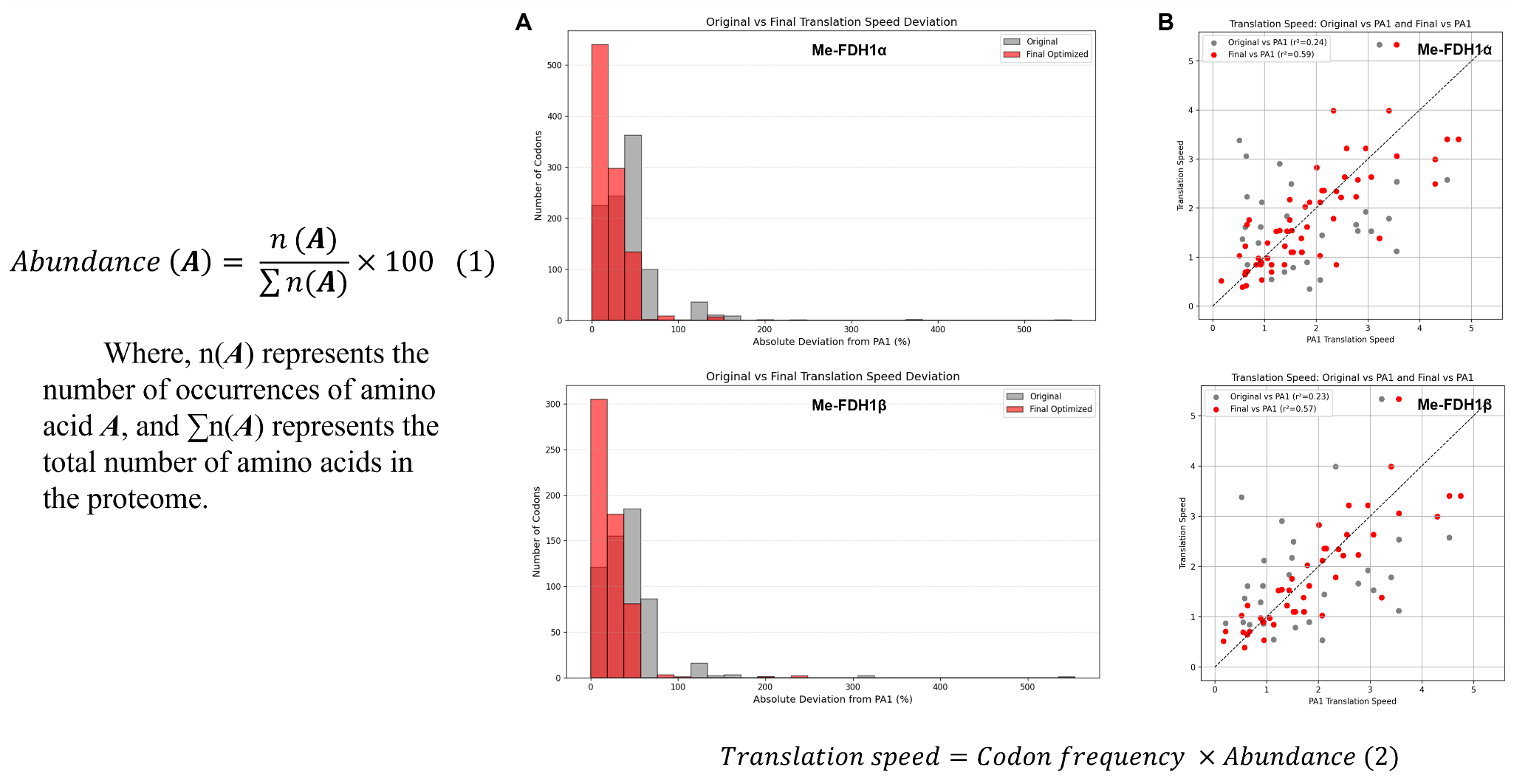
*

**Figure S2. Analysis of translation speed deviation and amino acid abundance in codon optimization.** (1) Equation for amino acid abundance. (2) Equation for translation speed. (**A**), (**B**) Comparison of translation speed deviation before and after codon optimization.


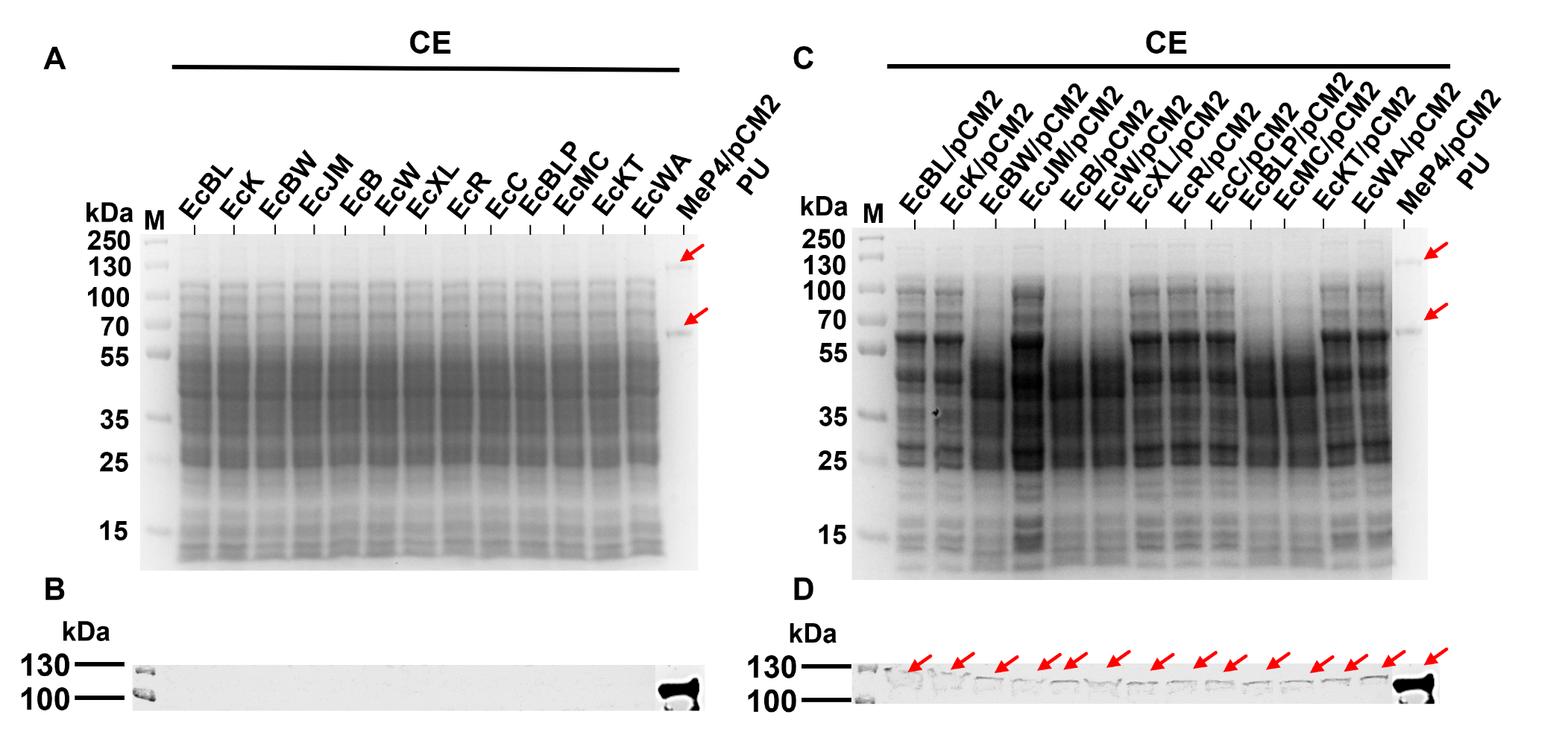


**Figure S3. Expression comparison of Me-FDH1 in various *E. coli*.** (**A**) SDS-PAGE analysis of wild type; (**B**) Western blot analysis of the wild-type sample using the same lane order as in panel (**A**); (**C**) SDS-PAGE analysis of various recombinant *E. coli* strains; (**D**) Western blot analysis of the wild-type sample using the same lane order as in panel (**C**). Lane M, protein ladder; Lane CE, crude cell extract; Lane PU, purified protein. Red arrows indicate the band corresponding to alpha (108 kDa) and beta (62 kDa) subunits.


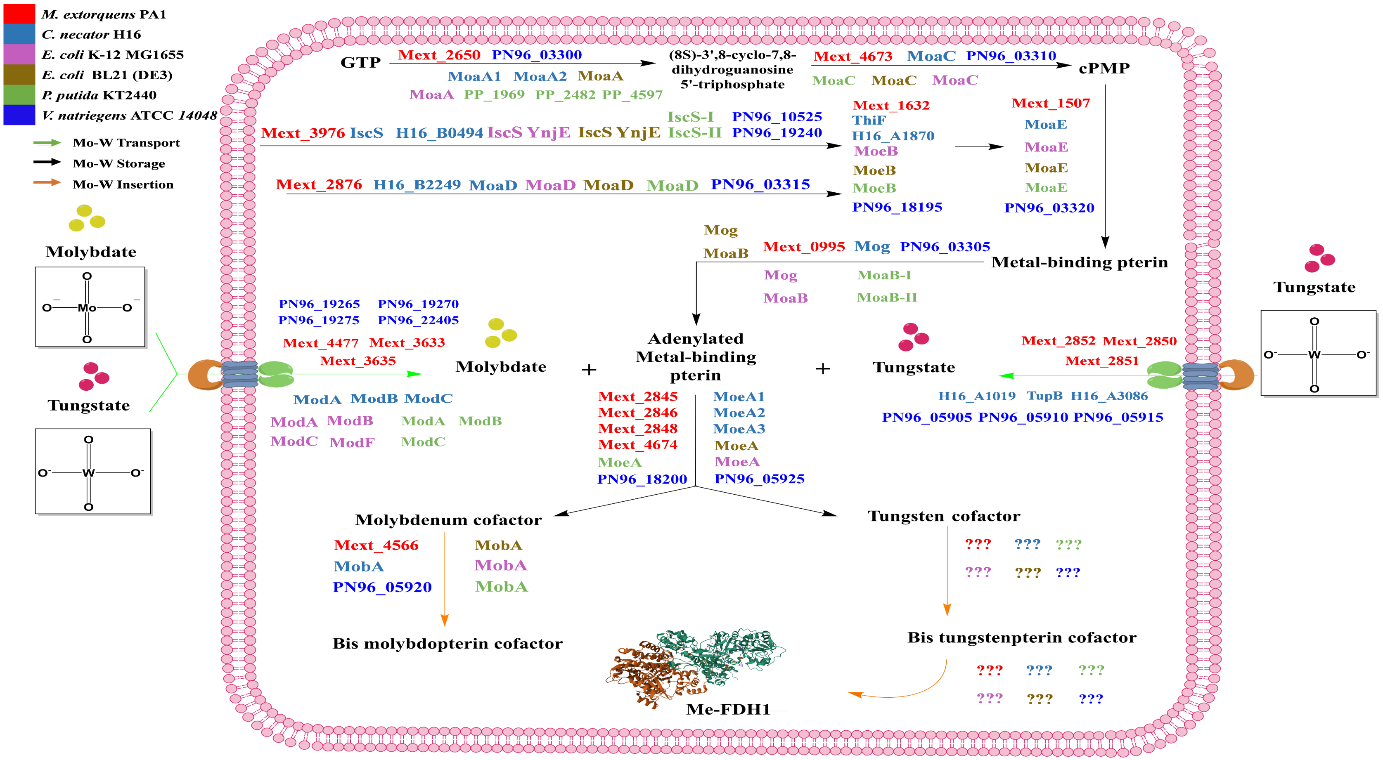
**Figure S4. Comparative analysis of putative W-bis-MGD biosynthesis pathways.** GTP, Guanosine 5'-triphosphate; cPMP, Cyclic pyranopterin monophosphate; MoaA1, molybdenum cofactor biosynthesis protein A; MoaC, molybdenum cofactor biosynthesis protein C; IscS, aminotransferase class V; YnjE, molybdopterin synthase sulfurtransferase;MoaD, molybdopterin converting factor subunit 1; ThiF, UBA/THIF-type NAD/FAD binding protein; MoeB, molybdopterin-synthase adenylyltransferase; MoaE, molybdopterin biosynthesis MoaE protein; Mog, molybdochelatase incorporating molybdenum into molybdopterin; MoaB, molybdenum cofactor biosynthesis protein B; MoeA1, MoeA2, MoeA3, molybdopterin biosynthesis protein subunits 1, 2, 3; MobA, molybdenum cofactor guanylyltransferase; Mext_2852, extracellular solute-binding protein family 1, TupB; Mext_2850, binding-protein-dependent transport systems inner membrane component, TupC; Mext_2851, tungstate transport system ATP-binding protein, TupA; ModA, molybdate transport system substrate-binding protein; ModB, molybdate transport system permease protein; ModC, molybdate transport system ATP-binding protein; Me-FDH1, tungsten dependent formate dehydrogenase I from *M.* *extorquens* PA1.


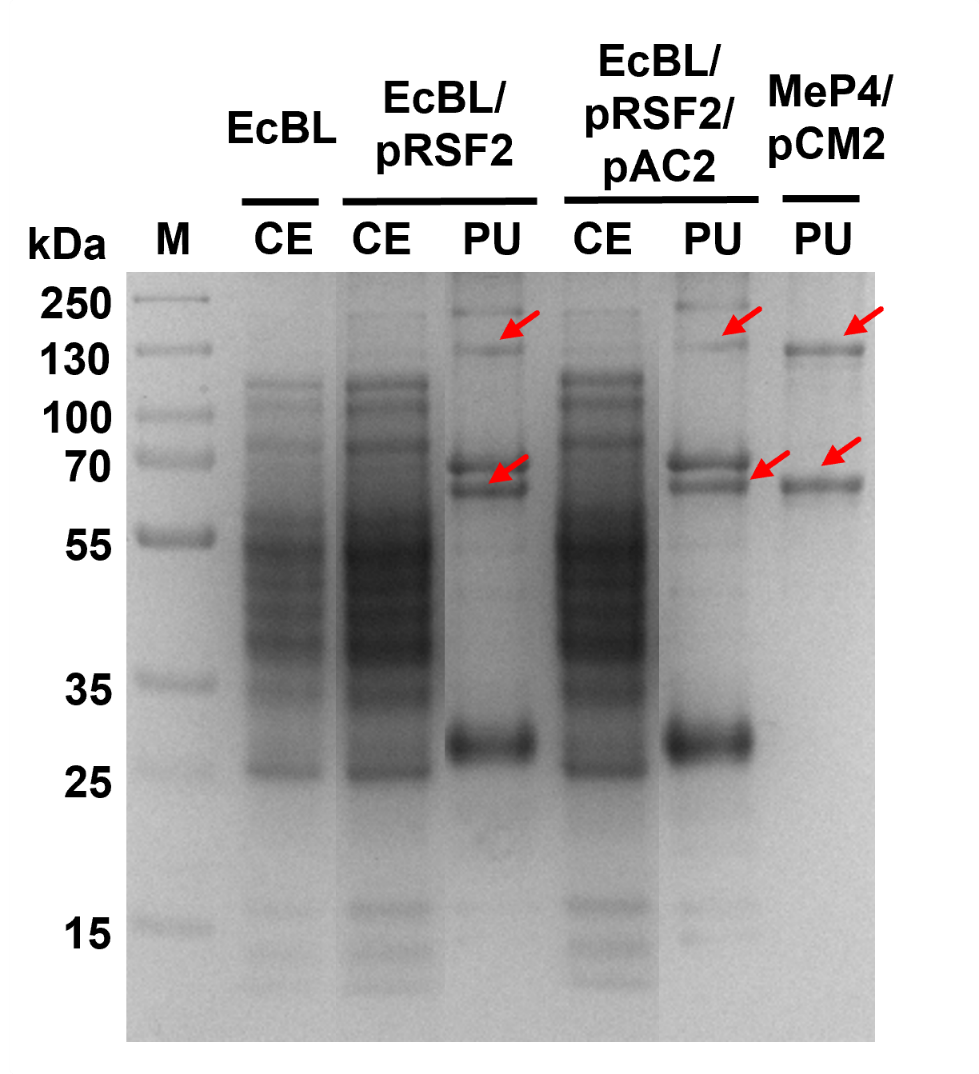


**Figure S5. Effect of tungstate transporter on Me-FDH1 expression in *E. coli* BL21 (DE3).** SDS-PAGE analysis of *E. coli* BL21 (DE3) host (EcBL). Lane M, protein ladder; Lane CE, crude cell extract; Lane PU, purified protein. Red arrows indicate the band corresponding to alpha (108 kDa) and beta (62 kDa) subunits.


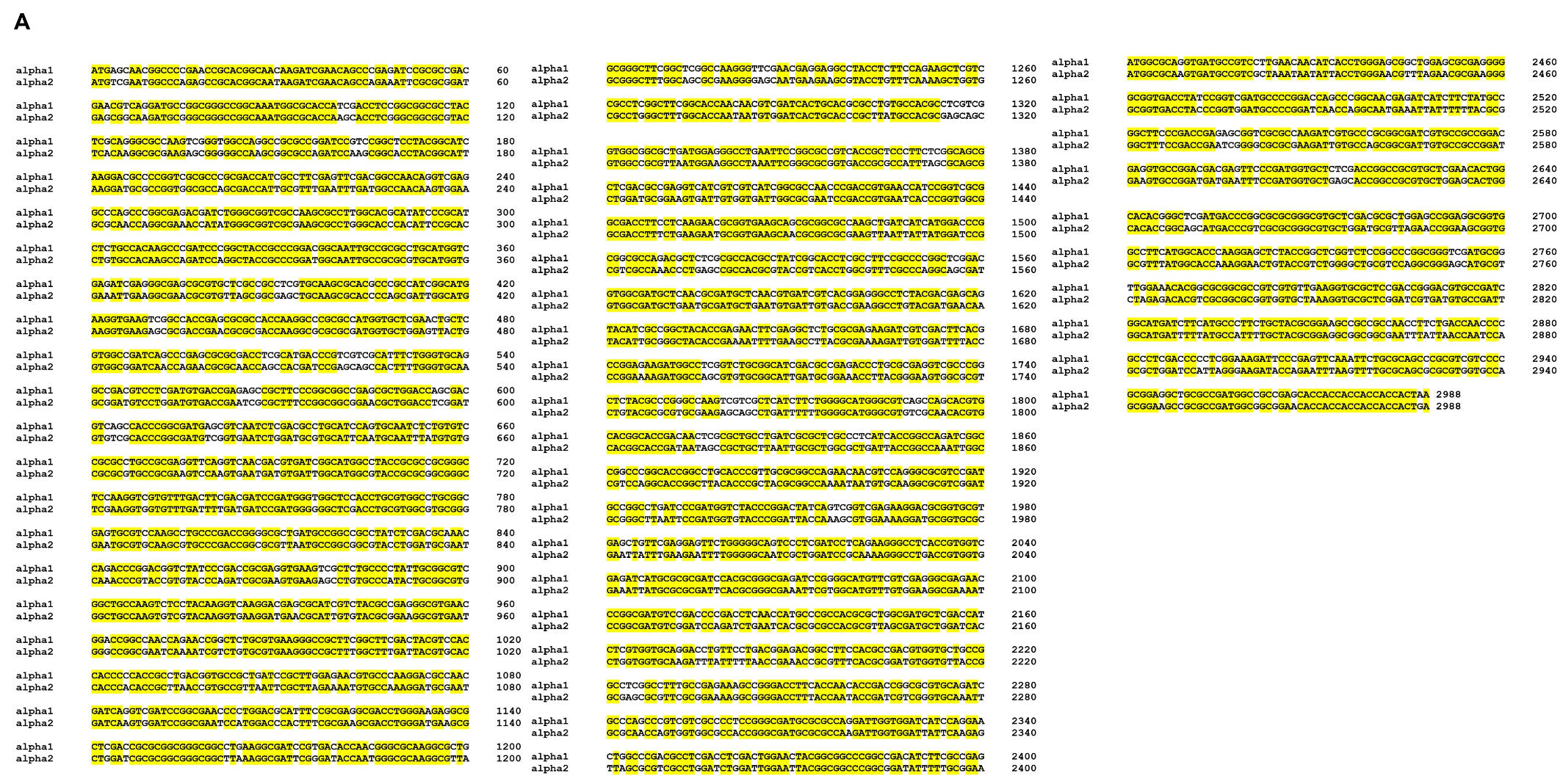


**
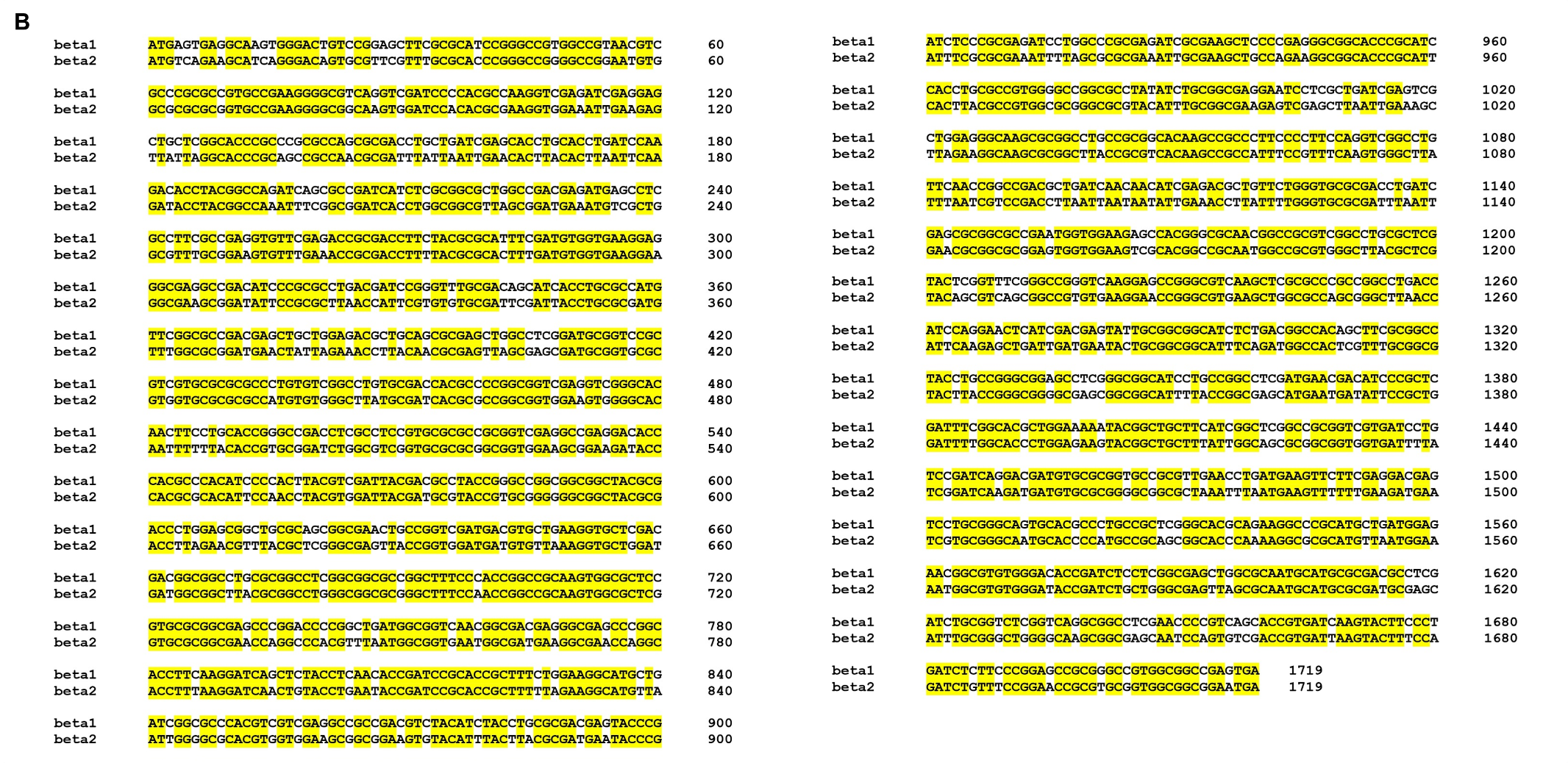
**

**Figure S6. Comparison of nucleotide sequences before and after codon optimization.** (**A**) alpha1, native alpha subunit sequence; alpha2, optimized alpha subunit sequence; Nucleotides identical between alpha1 and alpha2 are highlighted in yellow. (**B**) beta1, native beta subunit sequence; beta2, optimized beta subunit sequence; Nucleotides identical between beta1 and beta2 are highlighted in yellow.

# Supplementary Tables

**Table S1** Primers used in this study

| **Primers^a^** | **Description** | **Source** |
| --- | --- | --- |
| pCM3_P1 | GCCAGAGCCTGGAGCACTAGTATGAGCAACGGCCCCGAAC | This study |
| pCM3_P2 | GCTTTGTTAGCAGCCCTCGAGTTAGTGGTGGTGGTGGTGGTGC | This study |
| pCM3_P3 | CGGGCCGTGGCGGCCCTCGAGCGCCCAGCCCGACGATG | This study |
| pCM3_P4 | CGGTTCGGGGCCGTTACTAGTGCGTCCGCTCCAGGCTC | This study |
| pCM4_P1 | TCTCTGGCGGTGTTGACATGT | This study |
| pCM4_P2 | TCATATGTATATCTCCTTCTTAAAGTTAAACAAACTCTGGCATCGGGTGAGGAG | This study |
| pCM4_P3 | GAGATATACATATGAGCAACGGCCCCGAAC | This study |
| pCM4_P4 | CGAGCAGTTCGAGCACCATGGC | This study |
| pCM5_P1 | GCCGCAAGGAATGGTAAGCTTGATAAATTATCTCTGGCGGTGTTGACATGTG | This study |
| pCM5_P2 | ACTTGCCTCACTCATACTAGTATCTCCTTCTTAAAGTTAAACAAAGTCAGTGCGTC | This study |
| pCM5_P3 | GCCAGAGCCTGGAGCACTAGTATGAGCAACGGCCCCGAAC | This study |
| pCM5_P4 | AACGGATCTATCGATAAGCTTTAATGCGGTAGTTTATCACAG | This study |
| pCM6_P1 | GAATTAGCTTGCATGCCTGCAG | This study |
| pCM6_P2 | ACTTGCCTCACTCATACTAGTATCTCCTTCTTAAAG | This study |
| pCM6_P3 | CTTTAAGAAGGAGATACTAGTATGCACCACCACCACCACCACATGAGTGAGGCAAGTGGGAC | This study |
| pCM6_P4 | CGAGGCCAGCTCGCGCTGCAG | This study |
| pCM7_P1 | CAGGTCGACTCTAGATCACTGCCCG | This study |
| pCM7_P2 | TCATATGTATATCTCCTTCTTAAAGTTAAACAAAGTCAGTG | This study |
| pCM7_P3 | GAGATATACATATGAGCAACGGCCCCGAAC | This study |
| pCM7_P4 | CGAGCAGTTCGAGCACCATGGC | This study |
| pAC_MeTupBCA_P1 | ACAGAATTCGAGCTCGGTACCATGGTGCGCTCCGGGTGCG | This study |
| pAC_MeTupBCA_P2 | GCCGCTGTTCTTCCCGAGCGCGAAGAAGGGCTGAAAGCTTGGCTGTTTTGGCGGA | This study |
| pRSF-MeFDH1-invi-P1 | GACAAGCTTGCGGCCGCATAATG | This study |
| pRSF-MeFDH1-invi-P2 | GACCGTGTGCTTCTCAAATGCCTGAG | This study |
| pRSF-MeFDH1-invi-P3 | CGATGGTGTCCGGGATCTCGAC | This study |
| pRSF8-P1 | TAAGAAGGAGATATACATATGTCGAATGGCCCAGAGCCGCAC | This study |
| pRSF8-P2 | GGTTTCTTTACCAGACTCGAGTCAGTGGTGGTGGTGGTGGTGTT | This study |
| pET-P1 | AGAAGGAGATATACATATGTCAGAAGCATCAGGGAC | This study |
| pET-P2 | GGTTTCTTTACCAGACTCGAGTCAGTGGTGGTGGTGGTGGTGTT | This study |

^a^ Primer sequence is aligned from 5’ to 3’. Underlined nucleotides indicate restriction sites.

**Table S2** Comparative analysis of putative W-bis-MGD biosynthesis pathways

| **Reaction/ Pathway** | **Enzyme required for catalyzing reaction** | **Strain** | **Gene ID** | **Protein percent Identity (%)** |
| --- | --- | --- | --- | --- |
| GTP → (8S)-3’,8-cyclo-7,8-dihydroguanosine 5’-triphosphate | MoaA/ MoaA1/ MoaA2 | *Escherichia coli* BL21 (DE3) | MoaA | 32.41 |
|  |  | *Escherichia coli* str.K-12 substr. MG1655 | MoaA | 32.41 |
|  |  | *Methylorubrum extorquens* PA1 | Mext_2650 | 100 |
|  |  | *Cupriavidus necator* H16 | MoaA1 | 37.72 |
|  |  |  | MoaA2 | 34.81 |
|  |  | *Pseudomonas putida* KT2440 | PP_1969 | 32.61 |
|  |  |  | PP_2482 | 34.19 |
|  |  |  | PP_4597 | 48.79 |
|  |  | *V. natriegens* ATCC 14048 = KCTC 12726 | PN96_03300 | 32.10 |
| (8S)-3’,8-cyclo-7,8-dihydroguanosine 5’-triphosphate → Cyclic pyranopterin monophosphate (cPMP)/ Precursor Z | MoaC | *Escherichia coli* BL21 (DE3) | MoaC | 52.83 |
|  |  | *Escherichia coli* str.K-12 substr. MG1655 | MoaC | 52.83 |
|  |  | *Methylorubrum extorquens* PA1 | Mext_4673 | 100 |
|  |  | *Cupriavidus necator* H16 | MoaC | 57.23 |
|  |  | *Pseudomonas putida* KT2440 | MoaC | 55.77 |
|  |  | *V. natriegens* ATCC 14048 = KCTC 12726 | PN96_03310 | 55.97 |
| ***Sulfur relay system  Ubiquitin-like (1) → Ubiquitin-like-activating enzyme (E1) (2) → MoaE | IscS/ IscS-I/ IscS-II  (1) | *Escherichia coli* BL21 (DE3) | IscS | 36.15 |
|  |  | *Escherichia coli* str.K-12 substr. MG1655 | IscS | 36.15 |
|  |  | *Methylorubrum extorquens* PA1 | Mext_3976 | 100 |
|  |  | *Cupriavidus necator* H16 | IscS | 33.07 |
|  |  |  | H16_B0494 | 35.96 |
|  |  | *Pseudomonas putida* KT2440 | IscS-I | 32.98 |
|  |  |  | IscS-II | 35.75 |
|  |  | *V. natriegens* ATCC 14048 = KCTC 12726 | PN96_10525 | 34.04 |
|  |  |  | PN96_19240 | 36.55 |
|  | YnjE  (1) | *Escherichia coli* BL21 (DE3) | YnjE | 17.58 |
|  |  | *Escherichia coli* str.K-12 substr. MG1655 | YnjE | 17.58 |
|  |  | *Methylorubrum extorquens* PA1 | Mext_3976 | 100 |
|  |  | *Cupriavidus necator* H16 | H16_B0494 | 16.16 |
|  |  | *Pseudomonas putida* KT2440 | - | - |
|  |  | *V. natriegens* ATCC 14048 = KCTC 12726 | - | - |
|  | MoaD  (1) | *Escherichia coli* BL21 (DE3) | MoaD | 36.84 |
|  |  | *Escherichia coli* str.K-12 substr. MG1655 | MoaD | 35.53 |
|  |  | *Methylorubrum extorquens* PA1 | Mext_2876 | 100 |
|  |  | *Cupriavidus necator* H16 | H16_B2249 | 15.28 |
|  |  |  | MoaD | 45.78 |
|  |  | *Pseudomonas putida* KT2440 | MoaD | 30.38 |
|  |  | *V. natriegens* ATCC 14048 = KCTC 12726 | PN96_03315 | 31.25 |
|  | MoeB (2) | *Escherichia coli* BL21 (DE3) | MoeB | 42.17 |
|  |  | *Escherichia coli* str.K-12 substr. MG1655 | MoeB | 41.37 |
|  |  | *Methylorubrum extorquens* PA1 | Mext_1632 | 100 |
|  |  | *Cupriavidus necator* H16 | H16_A1870 | 27.02 |
|  |  |  | ThiF | 45.67 |
|  |  | *Pseudomonas putida* KT2440 | MoeB | 44.22 |
|  |  | *V. natriegens* ATCC 14048 = KCTC 12726 | PN96_18195 | 39.36 |
| Cyclic pyranopterin monophosphate (cPMP)/ Precursor Z → Metal-binding pterin | MoaE | *Escherichia coli* BL21 (DE3) | MoaE | 47.30 |
|  |  | *Escherichia coli* str.K-12 substr. MG1655 | MoaE | 47.30 |
|  |  | *Methylorubrum extorquens* PA1 | Mext_1507 | 100 |
|  |  | *Cupriavidus necator* H16 | MoaE | 46.94 |
|  |  | *Pseudomonas putida* KT2440 | MoaE | 48.63 |
|  |  | *V. natriegens* ATCC 14048 = KCTC 12726 | PN96_03320 | 43.24 |
| Tungstate specific transport | TupA | *Escherichia coli* BL21 (DE3) | - | - |
|  |  | *Escherichia coli* str.K-12 substr. MG1655 | - | - |
|  |  | *Methylorubrum extorquens* PA1 | Mext_2852 | 100 |
|  |  | *Cupriavidus necator* H16 | H16_A1019 | 45.76 |
|  |  | *Pseudomonas putida* KT2440 | - | - |
|  |  | *V. natriegens* ATCC 14048 = KCTC 12726 | PN96_05905 | 46.07 |
|  | TupB | *Escherichia coli* BL21 (DE3) | - | - |
|  |  | *Escherichia coli* str.K-12 substr. MG1655 | - | - |
|  |  | *Methylorubrum extorquens* PA1 | Mext_2850 | 100 |
|  |  | *Cupriavidus necator* H16 | TupB | 40.09 |
|  |  | *Pseudomonas putida* KT2440 | - | - |
|  |  | *V. natriegens* ATCC 14048 = KCTC 12726 | PN96_05910 | 32.33 |
|  | TupC | *Escherichia coli* BL21 (DE3) | - | - |
|  |  | *Escherichia coli* str.K-12 substr. MG1655 | - | - |
|  |  | *Methylorubrum extorquens* PA1 | Mext_2851 | 100 |
|  |  | *Cupriavidus necator* H16 | H16_A3086 | 34.63 |
|  |  | *Pseudomonas putida* KT2440 | - | - |
|  |  | *V. natriegens* ATCC 14048 = KCTC 12726 | PN96_05915 | 26.99 |
| Molybdate/ Tungstate non-specific transport | ModA | *Escherichia coli* BL21 (DE3) | - | - |
|  |  | *Escherichia coli* str.K-12 substr. MG1655 | ModA | 46.88 |
|  |  | *Methylorubrum extorquens* PA1 | Mext_4477 | 100 |
|  |  | *Cupriavidus necator* H16 | ModA | 36.51 |
|  |  | *Pseudomonas putida* KT2440 | ModA | 23.58 |
|  |  | *V. natriegens* ATCC 14048 = KCTC 12726 | PN96_19265 | 18.11 |
|  | ModB | *Escherichia coli* BL21 (DE3) | - | - |
|  |  | *Escherichia coli* str.K-12 substr. MG1655 | ModB | 60.69 |
|  |  | *Methylorubrum extorquens* PA1 | Mext_3633 | 100 |
|  |  | *Cupriavidus necator* H16 | ModB | 45.09 |
|  |  | *Pseudomonas putida* KT2440 | ModB | 28.83 |
|  |  | *V. natriegens* ATCC 14048 = KCTC 12726 | PN96_19270 | 31.19 |
|  | ModC | *Escherichia coli* BL21 (DE3) | - | - |
|  |  | *Escherichia coli* str.K-12 substr. MG1655 | ModC | 42.49 |
|  |  | *Methylorubrum extorquens* PA1 | Mext_3635 | 100 |
|  |  | *Cupriavidus necator* H16 | ModC | 37.72 |
|  |  | *Pseudomonas putida* KT2440 | ModC | 51.27 |
|  |  | *V. natriegens* ATCC 14048 = KCTC 12726 | PN96_19275 | 39.37 |
|  | ModF | *Escherichia coli* BL21 (DE3) | - | - |
|  |  | *Escherichia coli* str.K-12 substr. MG1655 | ModF | 100 |
|  |  | *Methylorubrum extorquens* PA1 | - | - |
|  |  | *Cupriavidus necator* H16 | - | - |
|  |  | *Pseudomonas putida* KT2440 | - | - |
|  |  | *V. natriegens* ATCC 14048 = KCTC 12726 | PN96_22405 | 37.68 |
| Metal-binding pterin → Adenylated Metal-binding pterin | MoaB/ MoaB-I/ MoaB-II | *Escherichia coli* BL21 (DE3) | MoaB | 47.06 |
|  |  | *Escherichia coli* str.K-12 substr. MG1655 | MoaB | 47.65 |
|  |  | *Methylorubrum extorquens* PA1 | Mext_0995 | 100 |
|  |  | *Cupriavidus necator* H16 | - | - |
|  |  | *Pseudomonas putida* KT2440 | MoaB-I | 51.40 |
|  |  |  | MoaB-II | 52.91 |
|  |  | *V. natriegens* ATCC 14048 = KCTC 12726 | PN96_03305 | 47.65 |
|  | Mog | *Escherichia coli* BL21 (DE3) | Mog | 27.54 |
|  |  | *Escherichia coli* str.K-12 substr. MG1655 | Mog | 27.54 |
|  |  | *Methylorubrum extorquens* PA1 | Mext_0995 | 100 |
|  |  | *Cupriavidus necator* H16 | Mog | 26.37 |
|  |  | *Pseudomonas putida* KT2440 | - | - |
|  |  | *V. natriegens* ATCC 14048 = KCTC 12726 | PN96_03305 | 47.65 |
| Adenylated Metal-binding pterin → Molybdenum cofactor | MoeA/ MoeA1/ MoeA2/ MoeA3 | *Escherichia coli* BL21 (DE3) | MoeA | 25.14 |
|  |  | *Escherichia coli* str.K-12 substr. MG1655 | MoeA | 25.42 |
|  |  | *Methylorubrum extorquens* PA1 | Mext_2845 | 100 |
|  |  |  | Mext_2846 | 28.97 |
|  |  |  | Mext_2848 | 27.97 |
|  |  |  | Mext_4674 | 27.71 |
|  |  | *Cupriavidus necator* H16 | MoeA1 | 24.78 |
|  |  |  | MoeA2 | 25.35 |
|  |  |  | MoeA3 | 28.16 |
|  |  | *Pseudomonas putida* KT2440 | MoeA | 24.07 |
|  |  | *V. natriegens* ATCC 14048 = KCTC 12726 | PN96_18200 | 23.10 |
|  |  |  | PN96_05925 | 19.49 |
| Molybdenum cofactor → Bis molybdopterin cofactor | MobA | *Escherichia coli* BL21 (DE3) | MobA | 34.90 |
|  |  | *Escherichia coli* str.K-12 substr. MG1655 | MobA | 34.90 |
|  |  | *Methylorubrum extorquens* PA1 | Mext_4566 | 100 |
|  |  | *Cupriavidus necator* H16 | MobA | 38.54 |
|  |  | *Pseudomonas putida* KT2440 | MobA | 29.79 |
|  |  | *V. natriegens* ATCC 14048 = KCTC 12726 | PN96_05920 | 30.05 |

***To synthesize these proteins, subsequent steps require additional proteins functioning as “auxiliary”, “helper”, or “carrier proteins”.

# Supplementary Text

The nucleotide sequences generated in this study have been deposited in NCBI GenBank under accession numbers PX353735 (codon optimized sequence *-* fdh1a) and PX353736 (codon optimized sequence *- fdh1*b).

**Supplementary Text S1. Codon-optimized sequence of the α-subunit - NCBI GenBank under accession numbers PX353735**

ATGTCGAATGGCCCAGAGCCGCACGGCAATAAGATCGAACAGCCAGAAATTCGCGCGGATGAGCGGCAAGATGCGGGCGGGCCGGCAAATGGCGCACCAAGCACCTCGGGCGGCGCGTACTCACAAGGCGCGAAGAGCGGGGGCCAAGCGGCGCCAGATCCAAGCGGCACCTACGGCATTAAGGATGCGCCGGTGGCGCCAGCGACCATTGCGTTTGAATTTGATGGCCAACAAGTGGAAGCGCAACCAGGCGAAACCATATGGGCGGTCGCGAAGCGCCTGGGCACCCACATTCCGCACCTGTGCCACAAGCCAGATCCAGGCTACCGCCCGGATGGCAATTGCCGCGCGTGCATGGTGGAAATTGAAGGCGAACGCGTGTTAGCGGCGAGCTGCAAGCGCACCCCAGCGATTGGCATGAAGGTGAAGAGCGCGACCGAACGCGCGACCAAGGCGCGCGCGATGGTGCTGGAGTTACTGGTGGCGGATCAACCAGAACGCGCAACCAGCCACGATCCGAGCAGCCACTTTTGGGTGCAAGCGGATGTCCTGGATGTGACCGAATCGCGCTTTCCGGCGGCGGAACGCTGGACCTCGGATGTGTCGCACCCGGCGATGTCGGTGAATCTGGATGCGTGCATTCAATGCAATTTATGTGTGCGCGCGTGCCGCGAAGTCCAAGTGAATGATGTGATTGGCATGGCGTACCGCGCGGCGGGCTCGAAGGTGGTGTTTGATTTTGATGATCCGATGGGGGGCTCGACCTGCGTGGCGTGCGGGGAATGCGTGCAAGCGTGCCCGACCGGCGCGTTAATGCCGGCGGCGTACCTGGATGCGAATCAAACCCGTACCGTGTACCCAGATCGCGAAGTGAAGAGCCTGTGCCCATACTGCGGCGTGGGCTGCCAAGTGTCGTACAAGGTGAAGGATGAACGCATTGTGTACGCGGAAGGCGTGAATGGGCCGGCGAATCAAAATCGTCTGTGCGTGAAGGGCCGCTTTGGCTTTGATTACGTGCACCACCCACACCGCTTAACCGTGCCGTTAATTCGCTTAGAAAATGTGCCAAAGGATGCGAATGATCAAGTGGATCCGGCGAATCCATGGACCCACTTTCGCGAAGCGACCTGGGATGAAGCGCTGGATCGCGCGGCGGGCGGCTTAAAGGCGATTCGGGATACCAATGGGCGCAAGGCGTTAGCGGGCTTTGGCAGCGCGAAGGGGAGCAATGAAGAAGCGTACCTGTTTCAAAAGCTGGTGCGCCTGGGCTTTGGCACCAATAATGTGGATCACTGCACCCGCTTATGCCACGCGAGCAGCGTGGCCGCGTTAATGGAAGGCCTAAATTCGGGCGCGGTGACCGCGCCATTTAGCGCAGCGCTGGATGCGGAAGTGATTGTGGTGATTGGCGCGAATCCGACCGTGAATCACCCGGTGGCGGCGACCTTTCTGAAGAATGCGGTGAAGCAACGCGGCGCGAAGTTAATTATTATGGATCCGCGTCGCCAAACCCTGAGCCGCCACGCGTACCGTCACCTGGCGTTTCGCCCAGGCAGCGATGTGGCGATGCTGAATGCGATGCTGAATGTGATTGTGACCGAAGGCCTGTACGATGAACAATACATTGCGGGCTACACCGAAAATTTTGAAGCCTTACGCGAAAAGATTGTGGATTTTACCCCGGAAAAGATGGCCAGCGTGTGCGGCATTGATGCGGAAACCTTACGGGAAGTGGCGCGTCTGTACGCGCGTGCGAAGAGCAGCCTGATTTTTTGGGGCATGGGCGTGTCGCAACACGTGCACGGCACCGATAATAGCCGCTGCTTAATTGCGCTGGCGCTGATTACCGGCCAAATTGGCCGTCCAGGCACCGGCTTACACCCGCTACGCGGCCAAAATAATGTGCAAGGCGCGTCGGATGCGGGCTTAATTCCGATGGTGTACCCGGATTACCAAAGCGTGGAAAAGGATGCGGTGCGCGAATTATTTGAAGAATTTTGGGGGCAATCGCTGGATCCGCAAAAGGGCCTGACCGTGGTGGAAATTATGCGCGCGATTCACGCGGGCGAAATTCGTGGCATGTTTGTGGAAGGCGAAAATCCGGCGATGTCGGATCCAGATCTGAATCACGCGCGCCACGCGTTAGCGATGCTGGATCACCTGGTGGTGCAAGATTTATTTTTAACCGAAACCGCGTTTCACGCGGATGTGGTGTTACCGGCGAGCGCGTTCGCGGAAAAGGCGGGGACCTTTACCAATACCGATCGTCGGGTGCAAATTGCGCAACCAGTGGTGGCGCCACCGGGCGATGCGCGCCAAGATTGGTGGATTATTCAAGAGTTAGCGCGTCGCCTGGATCTGGATTGGAATTACGGCGGCCCGGCGGATATTTTTGCGGAAATGGCGCAAGTGATGCCGTCGCTAAATAATATTACCTGGGAACGTTTAGAACGCGAAGGGGCGGTGACCTACCCGGTGGATGCCCCGGATCAACCAGGCAATGAAATTATTTTTTACGCGGGCTTTCCGACCGAATCGGGGCGCGCGAAGATTGTGCCAGCGGCGATTGTGCCGCCGGATGAAGTGCCGGATGATGAATTTCCGATGGTGCTGAGCACCGGCCGCGTGCTGGAGCACTGGCACACCGGCAGCATGACCCGTCGCGCGGGCGTGCTGGATGCGTTAGAACCGGAAGCGGTGGCGTTTATGGCACCAAAGGAACTGTACCGTCTGGGGCTGCGTCCAGGCGGGAGCATGCGTCTAGAGACACGTCGCGGCGCGGTGGTGCTAAAGGTGCGCTCGGATCGTGATGTGCCGATTGGCATGATTTTTATGCCATTTTGCTACGCGGAGGCGGCGGCGAATTTATTAACCAATCCAGCGCTGGATCCATTAGGGAAGATACCAGAATTTAAGTTTTGCGCAGCGCGCGTGGTGCCAGCGGAAGCCGCGCCGATGGCGGCGGAATGA

**Supplementary Text S2. Codon-optimized sequence of the β-subunit - NCBI GenBank under accession numbers PX353736**

ATGTCAGAAGCATCAGGGACAGTGCGTTCGTTTGCGCACCCGGGCCGGGGCCGGAATGTGGCGCGCGCGGTGCCGAAGGGGCGGCAAGTGGATCCACACGCGAAGGTGGAAATTGAAGAGTTATTAGGCACCCGCAGCCGCCAACGCGATTTATTAATTGAACACTTACACTTAATTCAAGATACCTACGGCCAAATTTCGGCGGATCACCTGGCGGCGTTAGCGGATGAAATGTCGCTGGCGTTTGCGGAAGTGTTTGAAACCGCGACCTTTTACGCGCACTTTGATGTGGTGAAGGAAGGCGAAGCGGATATTCCGCGCTTAACCATTCGTGTGTGCGATTCGATTACCTGCGCGATGTTTGGCGCGGATGAACTATTAGAAACCTTACAACGCGAGTTAGCGAGCGATGCGGTGCGCGTGGTGCGCGCGCCATGTGTGGGCTTATGCGATCACGCGCCGGCGGTGGAAGTGGGGCACAATTTTTTACACCGTGCGGATCTGGCGTCGGTGCGCGCGGCGGTGGAAGCGGAAGATACCCACGCGCACATTCCAACCTACGTGGATTACGATGCGTACCGTGCGGGGGGCGGCTACGCGACCTTAGAACGTTTACGCTCGGGCGAGTTACCGGTGGATGATGTGTTAAAGGTGCTGGATGATGGCGGCTTACGCGGCCTGGGCGGCGCGGGCTTTCCAACCGGCCGCAAGTGGCGCTCGGTGCGCGGCGAACCAGGCCCACGTTTAATGGCGGTGAATGGCGATGAAGGCGAACCAGGCACCTTTAAGGATCAACTGTACCTGAATACCGATCCGCACCGCTTTTTAGAAGGCATGTTAATTGGGGCGCACGTGGTGGAAGCGGCGGAAGTGTACATTTACTTACGCGATGAATACCCGATTTCGCGCGAAATTTTAGCGCGCGAAATTGCGAAGCTGCCAGAAGGCGGCACCCGCATTCACTTACGCCGTGGCGCGGGCGCGTACATTTGCGGCGAAGAGTCGAGCTTAATTGAAAGCTTAGAAGGCAAGCGCGGCTTACCGCGTCACAAGCCGCCATTTCCGTTTCAAGTGGGCTTATTTAATCGTCCGACCTTAATTAATAATATTGAAACCTTATTTTGGGTGCGCGATTTAATTGAACGCGGCGCGGAGTGGTGGAAGTCGCACGGCCGCAATGGCCGCGTGGGCTTACGCTCGTACAGCGTCAGCGGCCGTGTGAAGGAACCGGGCGTGAAGCTGGCGCCAGCGGGCTTAACCATTCAAGAGCTGATTGATGAATACTGCGGCGGCATTTCAGATGGCCACTCGTTTGCGGCGTACTTACCGGGCGGGGCGAGCGGCGGCATTTTACCGGCGAGCATGAATGATATTCCGCTGGATTTTGGCACCCTGGAGAAGTACGGCTGCTTTATTGGCAGCGCGGCGGTGGTGATTTTATCGGATCAAGATGATGTGCGCGGGGCGGCGCTAAATTTAATGAAGTTTTTTGAAGATGAATCGTGCGGGCAATGCACCCCATGCCGCAGCGGCACCCAAAAGGCGCGCATGTTAATGGAAAATGGCGTGTGGGATACCGATCTGCTGGGCGAGTTAGCGCAATGCATGCGCGATGCGAGCATTTGCGGGCTGGGGCAAGCGGCGAGCAATCCAGTGTCGACCGTGATTAAGTACTTTCCAGATCTGTTTCCGGAACCGCGTGCGGTGGCGGCGGAATGA
