# Supplementary figures and images for: Heterologous production of tungsten-dependent formate dehydrogenase I from Methylorubrum extorquens in Escherichia coli reveals α-subunit maturation as the major bottleneck
Source: Bioresour Bioprocess. 2026 Jul 24;13(1):105. doi: 10.1186/s40643-026-01092-7 (PMC13396061; doi:10.1186/s40643-026-01092-7)

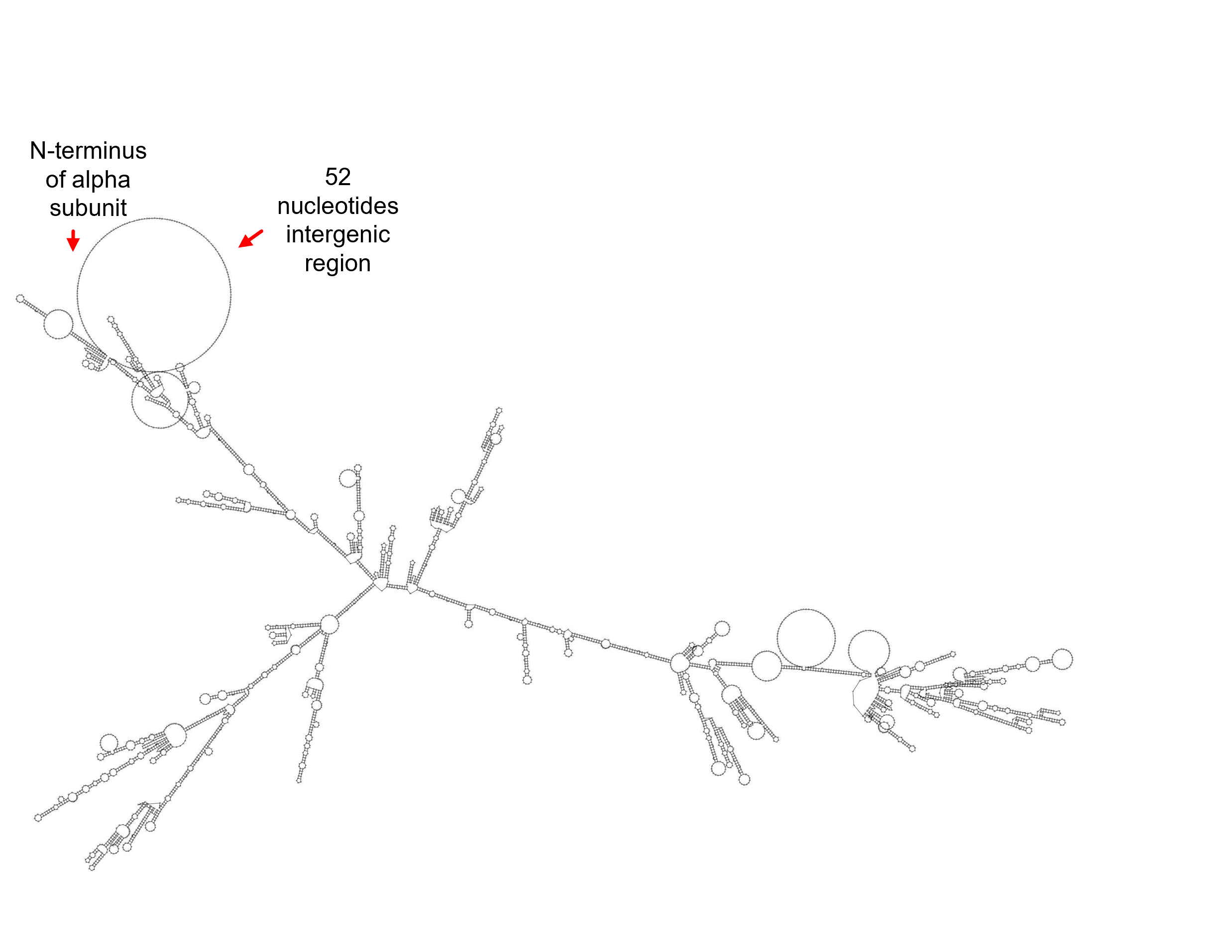

Supplement: Supplementary file 1 — Additional file 1: Word file containing Figures S1–S6, Tables S1–2, and supplementary text supporting the results of this study. [file 40643_2026_1092_MOESM1_ESM.zip › Supplementary Fig S1.png]

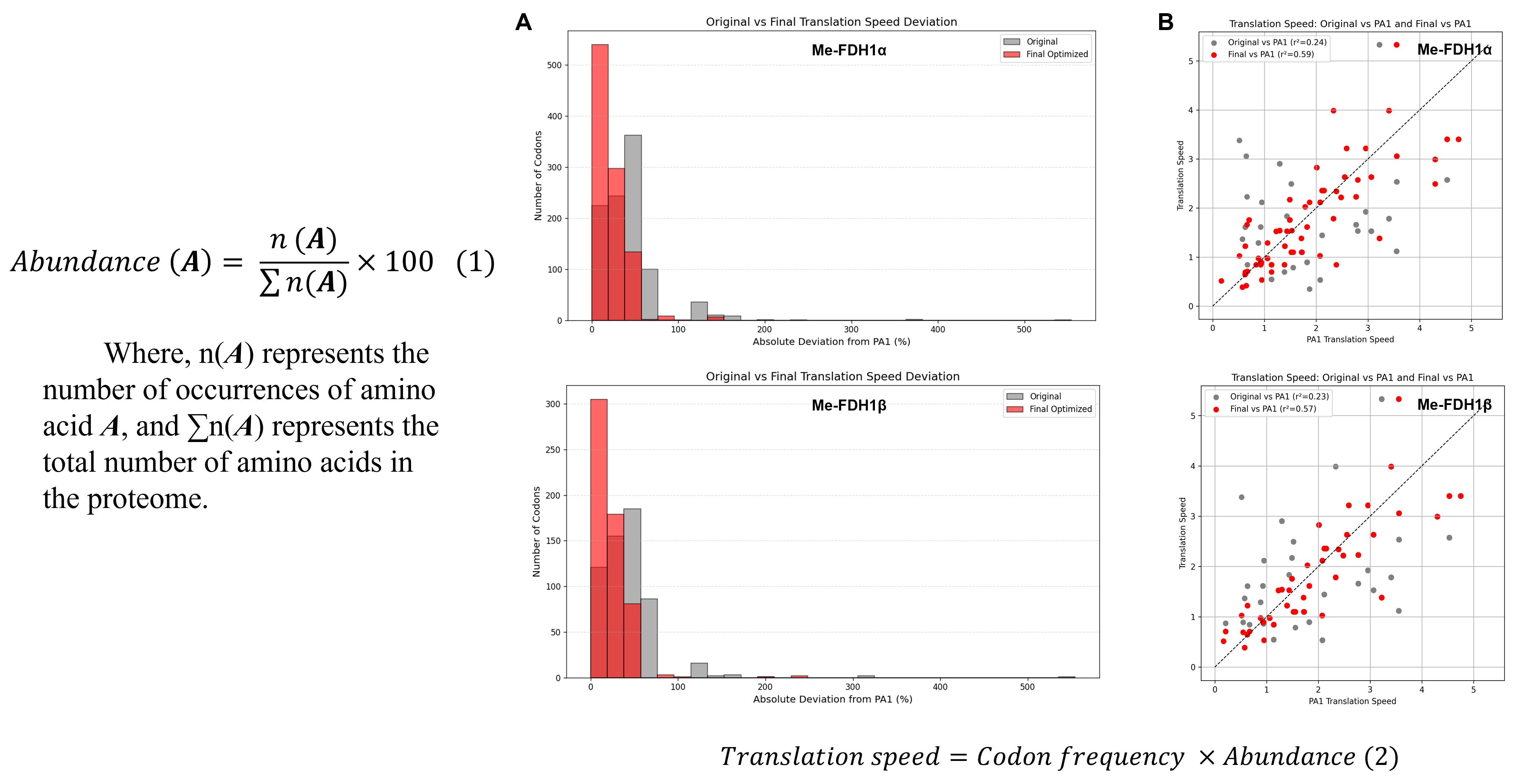

Supplement: Supplementary file 1 — Additional file 1: Word file containing Figures S1–S6, Tables S1–2, and supplementary text supporting the results of this study. [file 40643_2026_1092_MOESM1_ESM.zip › Supplementary Fig S2.png]

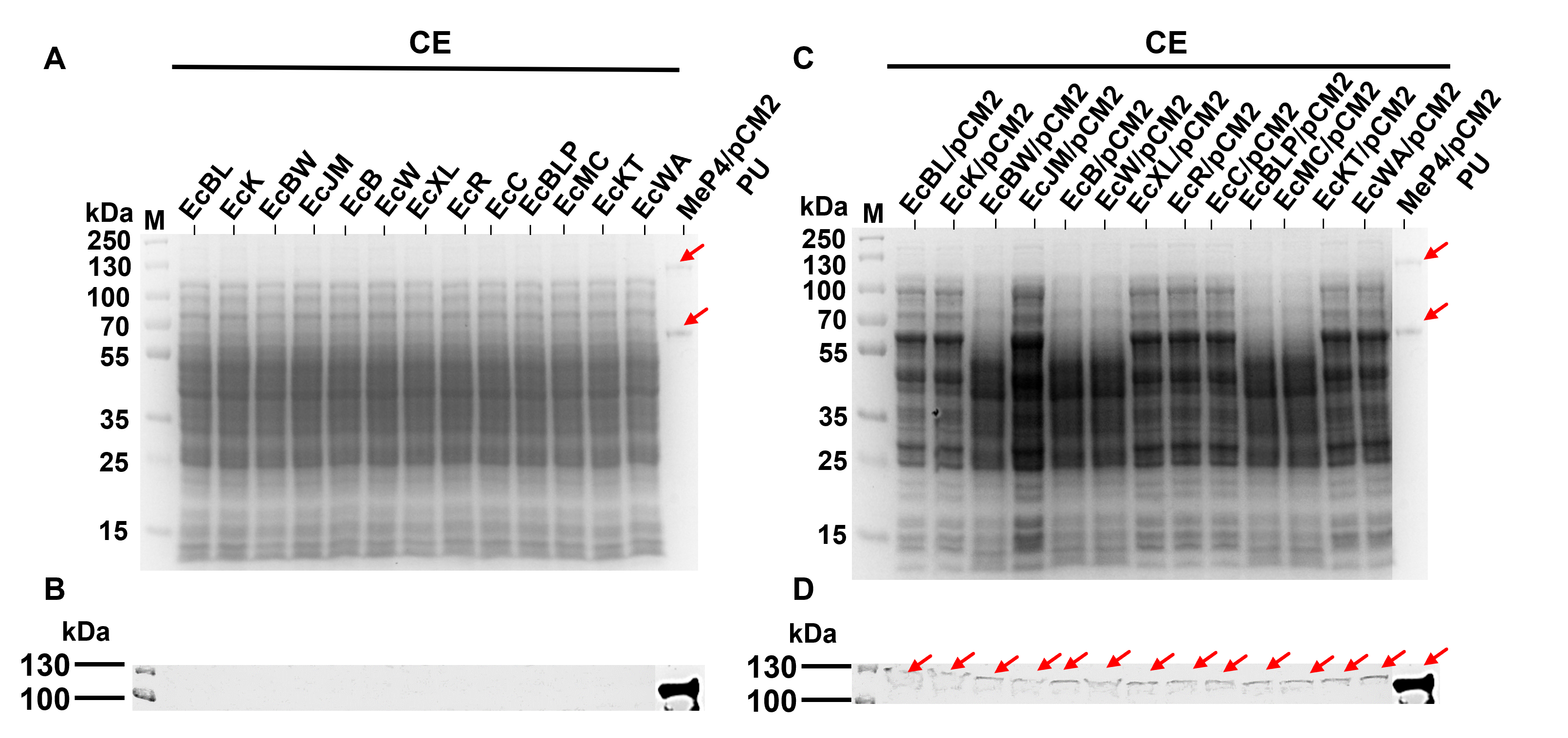

Supplement: Supplementary file 1 — Additional file 1: Word file containing Figures S1–S6, Tables S1–2, and supplementary text supporting the results of this study. [file 40643_2026_1092_MOESM1_ESM.zip › Supplementary Fig S3.png]

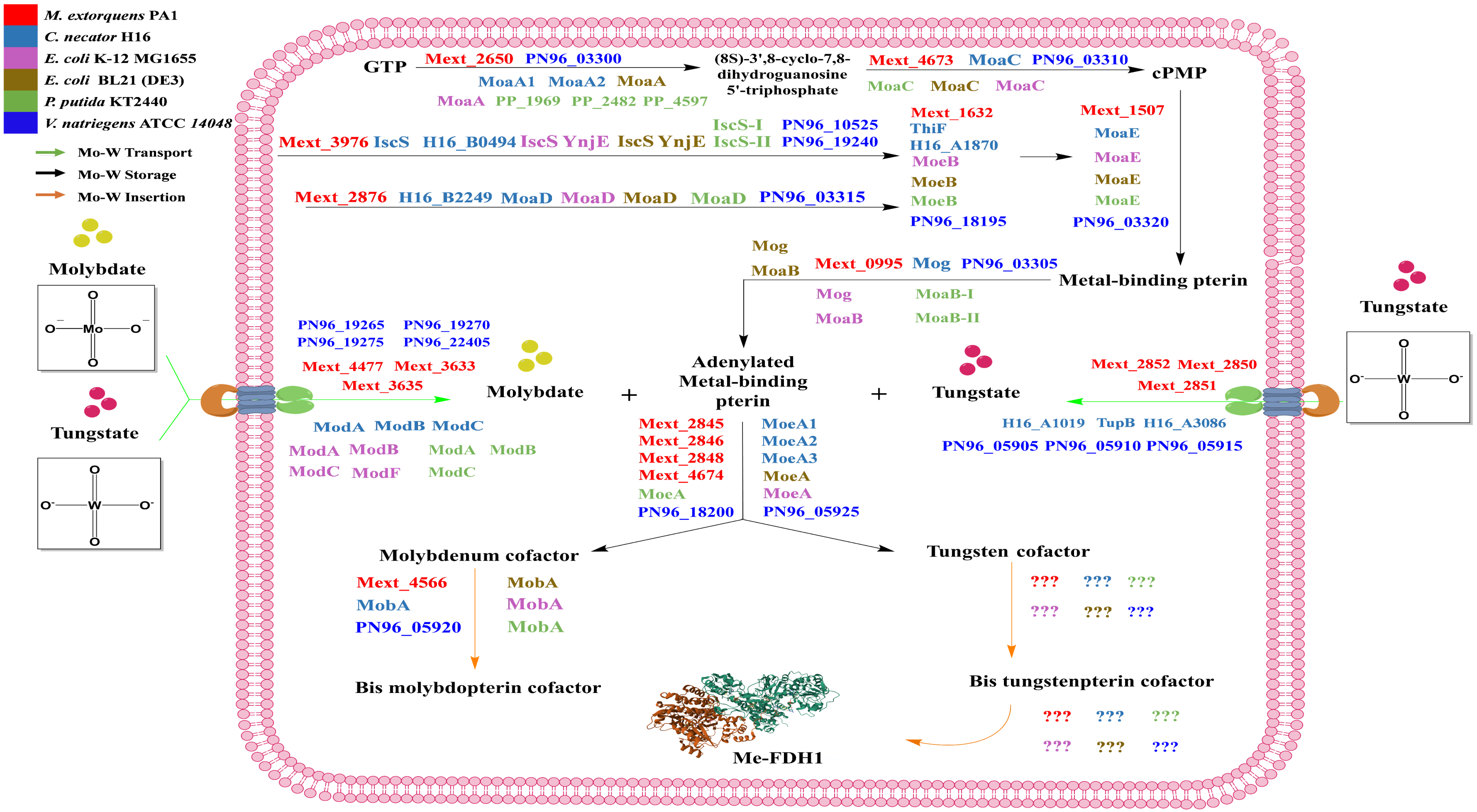

Supplement: Supplementary file 1 — Additional file 1: Word file containing Figures S1–S6, Tables S1–2, and supplementary text supporting the results of this study. [file 40643_2026_1092_MOESM1_ESM.zip › Supplementary Fig S4.png]

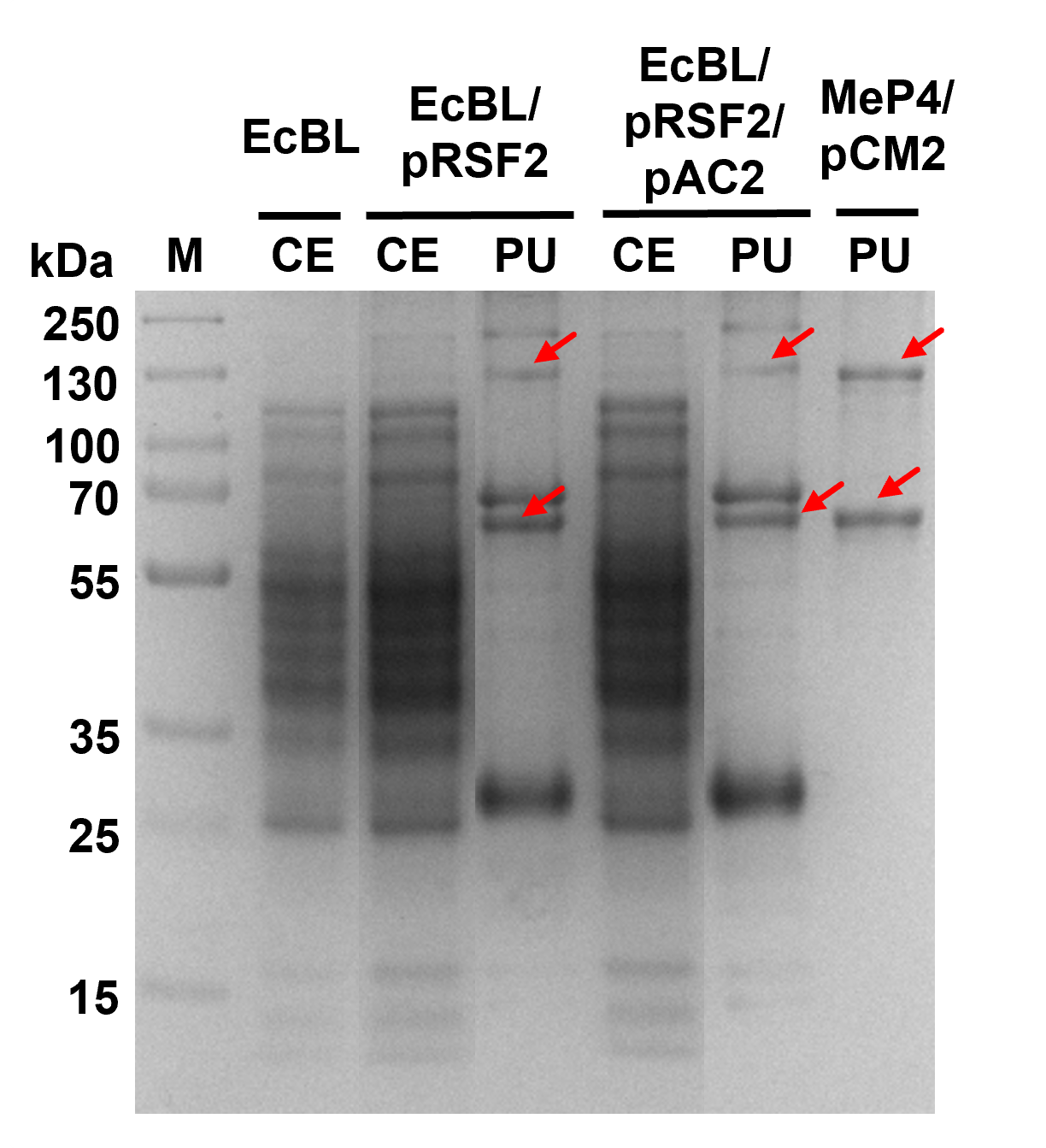

Supplement: Supplementary file 1 — Additional file 1: Word file containing Figures S1–S6, Tables S1–2, and supplementary text supporting the results of this study. [file 40643_2026_1092_MOESM1_ESM.zip › Supplementary Fig S5.png]

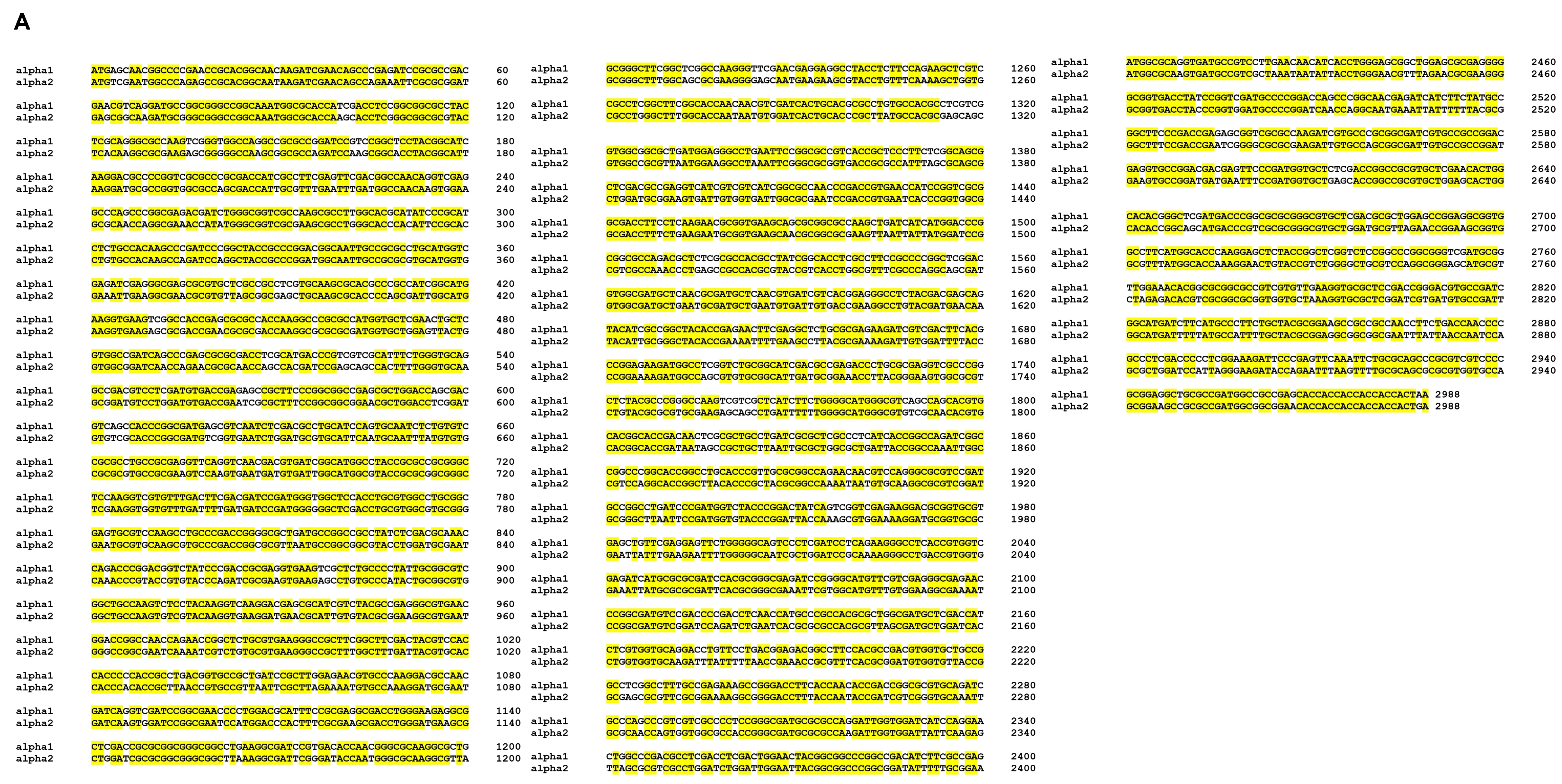

Supplement: Supplementary file 1 — Additional file 1: Word file containing Figures S1–S6, Tables S1–2, and supplementary text supporting the results of this study. [file 40643_2026_1092_MOESM1_ESM.zip › Supplementary Fig S6A.png]

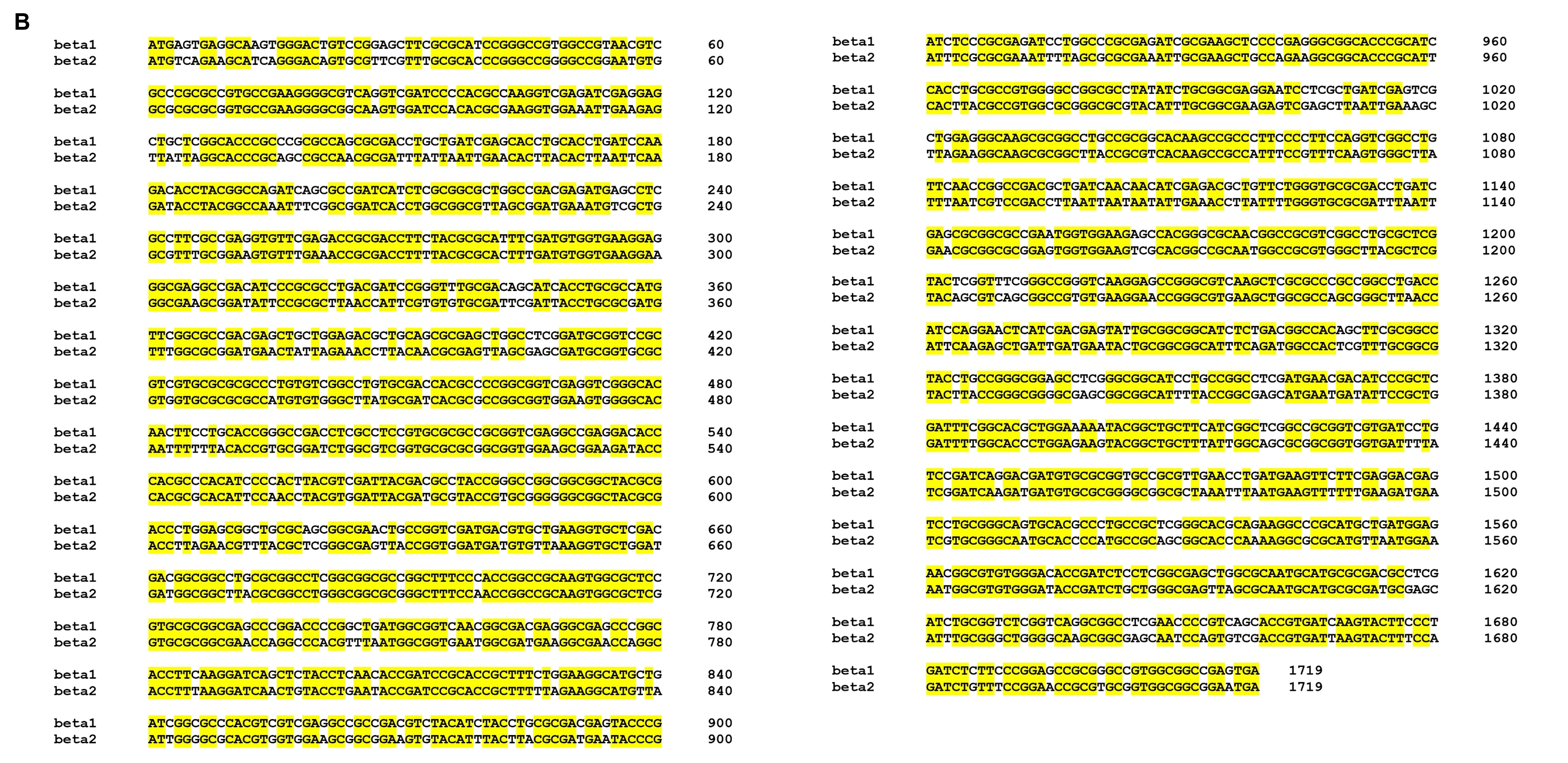

Supplement: Supplementary file 1 — Additional file 1: Word file containing Figures S1–S6, Tables S1–2, and supplementary text supporting the results of this study. [file 40643_2026_1092_MOESM1_ESM.zip › Supplementary Fig S6B.png]
