# Supplementary material for: Heterologous production of tungsten-dependent formate dehydrogenase I from Methylorubrum extorquens in Escherichia coli reveals α-subunit maturation as the major bottleneck
Source: Bioresour Bioprocess. 2026 Jul 24;13(1):105. doi: 10.1186/s40643-026-01092-7 (PMC13396061; doi:10.1186/s40643-026-01092-7)
Supplement: Supplementary file 1 — Additional file 1: Word file containing Figures S1–S6, Tables S1–2, and supplementary text supporting the results of this study. [file 40643_2026_1092_MOESM1_ESM.zip › Response to Reviewers.docx]

**Response to Reviewer’s Comments**

**Title:** Heterologous production of tungsten-dependent formate dehydrogenase I from *Methylorubrum extorquens* in *Escherichia coli* reveals α-subunit maturation as the major bottleneck

**Reference no:** BIOB-D-26-00105R1

We thank the Editor and the Reviewers for the positive assessment of our manuscript and for the opportunity to submit a revised version. Reviewer #4 recommended the addition of a table describing the different strains to help readers keep track of them. We have addressed this point as detailed below. The corresponding changes are highlighted in the revised manuscript.

**Response to the Editor.** We thank the Editor for handling our submission and for the opportunity to respond to the reviewers. All comments have been carefully addressed through text revision, expanded discussion, and clearer presentation of the data. A detailed point-by-point response is provided below, followed by the revised manuscript with tracked changes.

**Response to Reviewer #4 comment**

**Comment.** This article by Nguyen Ngoc Minh Chau and coworkers report on several attemps to overexpress the tungsten formate dehydrogenase from *M. extorquens* in *E. coli*. Though the attempts are only moderately successful, both in terms of quantity but also quality (specific activity), I find the article interesting and suitable for the readership of Bioresources and Bioprocessing. Indeed, the general trend now is to focus only on fully successful expressions, leading to a large amount of time wasted because groups repeat the (unpublished) mistakes of other groups. This article provides a first step into a better system for overexpressing the formate dehydrogenase. The fact that another group can do it is the interest of this article.

I find the current version suitable for publication, and I appreciate the responses to the other reviewers, so I don't see what really to add, excepted maybe a table describing the different strains, to help the reader remember what they are.

**Response.** We thank the reviewer for the positive evaluation of our manuscript and for this helpful suggestion.

The strains were already listed in Table 1 (“Plasmids and strains used in this study”; Line 768); however, the original entries gave only the strain name, which did not convey what distinguishes each strain. We have now expanded the Description column for every strain so that each entry states its relevant genotype or engineering feature and, where applicable, the specific experiment in which it was used. This allows the reader to recall the identity and purpose of each strain directly from Table 1 without returning to the Results text. The revised strain section of Table 1 now reads as follows:

**Table 1** Plasmids and strains used in this study

| **Plasmids** | **Description** | **Source** |
| --- | --- | --- |
| pCM2 | pCM110-P_L/O4_-RBS_b_-fdh1ba-His_6_-T7_ter_, Tet^R^ | (Ryu et al., 2024) |
| pCM3 | pCM110-P_L/O4_- RBS_b_- fdh1a-His_6_- fdh1b-T7_ter_, Tet^R^ | This study |
| pCM4 | pCM110-P_L/O4_- RBS_b_- fdh1b- RBS_b_-fdh1a-His_6-_T7_ter_, Tet^R^ | This study |
| pCM5 | pCM110-P_L/O4_- RBS_b_-His_6_- fdh1b-T7_ter_-P_L/O4_- RBS_b_- fdh1a-His_6_-T7_ter_, Tet^R^ | This study |
| pCM6 | pCM110-P_L/O4_- RBS_b_-His6- fdh1b-T7_ter_, Tet^R^ | This study |
| pCM7 | pCM110-P_L/O4_- RBS_b_- fdh1a-His6-T7_ter_, Tet^R^ | This study |
| pAC1 | pACYC184, p15A ColE1 ori, Cm^R^, Tet^R^ | Addgene |
| pAC2 | pAC-P_tac_-RBS-tupBCA-T7_ter_, Cm^R^ | This study |
| pRSF1 | pRSF-Duet-1, RSF1030 ori, P_T7_, Kan^R^ | Addgene |
| pRSF2 | pRSF-P_T7_-fdh1ba-His_6_-T7_ter_, Kan^R^ | This study |
| pRSF3 | pRSF-P_T7_- fdh1a-His_6_-T7_ter_, Kan^R^ | This study |
| pRSF4 | pRSF-P_T7_- fdh1b-His_6_-T7_ter_, Kan^R^ | This study |
| pRSF5 | pRSF-P_T7_- fdh1b-His_6_-T7_ter_-P_T7_- fdh1a-His_6_-T7_ter_, Kan^R^ | This study |
| pRSF6 | pRSF-P_T7_-SUMO- fdh1ba-His_6_-T7_ter_, Kan^R^ | This study |
| pRSF7 | pRSF-P_T7_-SUMO- fdh1a-His_6_-T7_ter_, Kan^R^ | This study |
| pRSF8 | pRSF-P_T7_- fdh1a codon optimized-His_6_-T7_ter_, Kan^R^ | This study |
| pET1 | pET-Duet-1, pBR322-derived ColE1 ori, P_T7_, Am^R^ | Addgene |
| pET2 | pET-P_T7_- fdh1b codon optimized-His_6_-T7_ter_, Am^R^ | This study |
| pG-KJE8 | P_araBAD_-dnaK-dnaJ-grpE, P_lac_-groES-groEL, Cm^R^, p15A ori | TaKaRa, Japan |
| pG-Tf2 | P_lac_-tig, P_lac_-groES-groEL, Cm^R^, p15A ori | TaKaRa, Japan |
| **Strains** | **Description** | **Source** |
| EcB | E. coli B wildtype; B-lineage strain included in the host-background screen | DSMZ |
| EcBL | E. coli BL21 (DE3) wildtype; B-derived T7 expression host carrying λDE3, deficient in Lon and OmpT proteases; also used for TupBCA transporter complementation | (Nguyen-Vo et al., 2020) |
| EcBLP | E. coli BL21 Star™ (DE3) pLysS wildtype; T7 expression host carrying rne131 for enhanced mRNA stability and pLysS for basal-expression repression | New England Biolabs |
| EcBW | E. coli BW25113 wildtype; K-12 derivative and parent strain of the Keio knockout collection | (Sundara Sekar et al., 2016) |
| EcC | E. coli C ATCC8739 wildtype; C-lineage strain included in the host-background screen | (Sundara Sekar et al., 2016) |
| E. coli DH5α | Cloning host | Toyobo, Japan |
| EcJM | E. coli JM109 (DE3) wildtype; K-12 derivative carrying λDE3 for T7-driven expression | Promega, Korea |
| EcK | E. coli K-12 MG1655 wildtype; standard K-12 reference strain used for initial expression, operon-architecture analysis, and chaperone co-expression tests | (Nguyen-Vo et al., 2020) |
| EcKT | E. coli K-12 Shuffle® T7 wildtype; redox-engineered T7-compatible strain with ΔtrxB/Δgor and cytoplasmic DsbC, used for SUMO-fusion and codon-harmonization tests | New England Biolabs |
| EcMC | E. coli MC1061 wildtype; K-12 derivative previously used for heterologous expression of the Mo-dependent Rhodobacter capsulatus FDH | Thermo Fisher Scientific; (Böhmer et al., 2014; Hartmann and Leimkühler, 2013) |
| EcWA | E. coli W ATCC 9637 wildtype; W-lineage strain included in the host-background screen | (Nguyen-Vo et al., 2020) |
| EcW | E. coli W3110 wildtype; K-12-lineage strain included in the host-background screen | ATCC |
| EcXL | E. coli XL-1 Blue wildtype; K-12 derivative with cloning-related recA1 and endA1 background | New England Biolabs |
| EcR | E. coli Rosetta 2 (DE3) wildtype; BL21(DE3) derivative supplying rare-codon tRNAs (argU, argW, ileX, glyT, leuW, proL) via pRARE2 | Novagen |
| MeP | M. extorquens PA1, fdh1 gene source | DSMZ, Germany |
| MeP4 | MePΔfdh1Δfdh2Δfdh3Δfdh4; homologous expression host used for operon-architecture comparison | (Ryu et al., 2024) |

**Reference**

Hartmann, T., Leimkühler, S., 2013. The oxygen-tolerant and NAD+-dependent formate dehydrogenase from Rhodobacter capsulatus is able to catalyze the reduction of CO2 to formate. FEBS Journal 280, 6083–6096. https://doi.org/10.1111/febs.12528

Böhmer, N., Hartmann, T., Leimkühler, S., 2014. The chaperone FdsC for Rhodobacter capsulatus formate dehydrogenase binds the bis-molybdopterin guanine dinucleotide cofactor. FEBS Lett. 588, 531–537. https://doi.org/10.1016/j.febslet.2013.12.033
